# The Epstein-Barr Virus Oncoprotein, LMP1, Regulates the Function of SENP2, a SUMO-protease

**DOI:** 10.1038/s41598-019-45825-5

**Published:** 2019-07-02

**Authors:** Thomas L. Selby, Natalie Biel, Matthew Varn, Sheetal Patel, Akash Patel, Leslie Hilding, Ashley Ray, Tabithia Ross, Wyatt T. Cramblet, C. Randall Moss, Angela J. Lowrey, Gretchen L. Bentz

**Affiliations:** Division of Biomedical Sciences, Mercer University School of Medicine, Macon, Georgia USA

**Keywords:** Sumoylation, Virus-host interactions, Tumour virus infections

## Abstract

Epstein-Barr virus (EBV) latent membrane protein-1 (LMP1) activates numerous signal transduction pathways using its C-terminal activating regions. We reported that LMP1 increased global levels of sumoylated proteins, which aided the oncogenic nature of LMP1. Because increased protein sumoylation is detected in numerous cancers, we wanted to elucidate additional mechanisms by which LMP1 modulates the sumoylation machinery. Results indicated that SUMO-protease activity decreased in a LMP1-dependent manner, so we hypothesized that LMP1 inhibits SUMO-protease activity, resulting in reduced de-sumoylation of cellular proteins, which contributes to the detected accumulation of sumoylated proteins in EBV-positive lymphomas. Focusing on SENP2, findings revealed that LMP1 expression corresponded with increased sumoylation of SENP2 at K48 and K447 in a CTAR-dependent manner. Interestingly, independent of LMP1-induced sumoylation of SENP2, LMP1 also decreased SENP2 activity, decreased SENP2 turnover, and altered the localization of SENP2, which led us to investigate if LMP1 regulated the biology of SENP2 by a different post-translational modification, specifically ubiquitination. Data showed that expression of LMP1 inhibited the ubiquitination of SENP2, and inhibition of ubiquitination was sufficient to mimic LMP1-induced changes in SENP2 activity and trafficking. Together, these findings suggest that LMP1 modulates different post-translational modifications of SENP2 in order to modulate its biology and identify a third member of the sumoylation machinery that is manipulated by LMP1 during latent EBV infections, which can affect oncogenesis.

## Introduction

The post-translational modification of proteins by small ubiquitin-like modifier (SUMO) is involved in multiple cellular processes, such as protein trafficking, protein stability, the ability of proteins to interact with other proteins or DNA, and protein function^1–3^. As a dynamic process, protein sumoylation starts with the transcription and translation of the *sumo* genes. The resulting pro-peptide (SUMO-1, -2, -3, and -4) undergoes maturation when a SUMO-protease cleaves the C-terminal tail of the pro-peptide and reveals the C-terminal SUMO di–glycine motif. Following activation by the SUMO-activating enzyme (SAE1/SAE2), SUMO is passed to the SUMO-conjugating enzyme (Ubc9), which mediates the isopeptide bond between SUMO and the lysine residue within the canonical sumoylation motif (ΨKxD/E)^4^. The whole process can be enhanced by a SUMO-E3 ligase^5,6^ and reversed by  Sentrin-specific proteases or SENPs^3^.

Dysregulation of cellular sumoylation processes is a feature of a variety of diseases^7–15^, and targeting Ubc9 and the SENP expression and function has been proposed as potential new therapeutic targets^9,16,17^. Sumoylation processes are important during lytic and latent Epstein-Barr virus (EBV) infections^18–26^. We identified a specific function of latent membrane protein-1 (LMP1) in the induction of the sumoylation of cellular proteins in order to modulate LMP1-induced cell migration and aid the maintenance of EBV latency^15,27^. LMP1 is the principal oncoprotein of EBV and is expressed in multiple EBV-associated lymphoid tumors^28,29^. LMP1 is a constitutively active integral membrane protein that mimics the tumor necrosis factor (TNF) receptor family members^30^. The cytoplasmic C-terminal region of LMP1 contains three C-terminal activating regions (CTARs)^30,31^. The majority of LMP1-induced signaling is through the extensively characterized CTAR1 and CTAR2 domains, but we first identified that the ability of LMP1 to dysregulate sumoylation processes was through CTAR3^15^. Specifically, LMP1 CTAR3 hijacks enzymatically active Ubc9 during latent EBV infections^15^, which results in increased sumoylation of cellular proteins^15^, aids LMP1-induced cell migration^15^, modulates innate immune responses^32^, and aids the maintenance of latency^27^. These findings prompted us to question if LMP1 targeted additional steps of the sumoylation processes and manipulated additional members of the sumoylation machinery. Preliminary data led us to focus on the SENPs.

There are six SENP isoforms (SENP1–3 and 5–7) that possess de-sumoylating activity^33^. These isoforms are divided into sub-families depending on their ability to mediate the maturation of SUMO pro-peptides and their specificity in de-sumoylating proteins modified by SUMO-1 or SUMO-2/3. The first sub-family consists of SENP1 and SENP2, and they are able to de-sumoylate SUMO-1-, -2-, and -3-modified proteins^33^. The remaining two SENP sub-families (SENP3 and SENP5 or SENP6 and SENP7) preferentially de-sumoylate SUMO-2/3-modified proteins over SUMO-1-modified proteins^33^. In addition, SENP1, SENP2, and SENP5 mediate the maturation of the SUMO pro-peptides^33^. Together, the SENPs are essential to the modulation of cellular sumoylation processes due to their ability to regulate intracellular pools of free SUMO and de-sumoylate modified proteins.

The C-terminal domain of the SENPs interfaces with SUMO, which allows the protease to interact with the SUMO pro-peptides or sumoylated proteins^34^, The C-terminal domain also contains the catalytic domain that consists of the catalytic triad (aspartic acid, histidine, and cysteine residues)^35,36^, which mediates SUMO maturation or protein de-sumoylation by recognizing and cleaving after the SUMO di-glycine motif ^35,36^. Because SENPs are critical for two steps of the sumoylation process, it is possible that they are targeted for abnormal regulation or expression during disease progression, such as viral infection. While it is known that SUMO-protease inhibitors can inhibit the replication of HIV^17^ and several viruses possibly encode mimics of cellular SENPs^37–39^, the ability of any virus to affect the function or expression of any SENP remains undocumented.

Here, we show that LMP1 modulates SENP2 expression and function. LMP1 expression increases the sumoylation of SENP2. Independent of this sumoylation, LMP1 also inhibits SENP2 activity, stabilizes SENP2 expression, and mediates the movement of SENP2 from within the nucleus to the outer nuclear envelope. Furthermore, LMP1 expression decreased the ubiquitination of SENP2, and inhibition of cellular ubiquitination processes was sufficient to modulate SENP2 activity and trafficking. Together, the end result is the decreased de-sumoylation of cellular proteins, which aids the documented global increase in sumoylated proteins during latent infections^40^.

## Results

### Cellular de-sumoylating activities were decreased in LMP1-expressing cells

**Figure 1:**
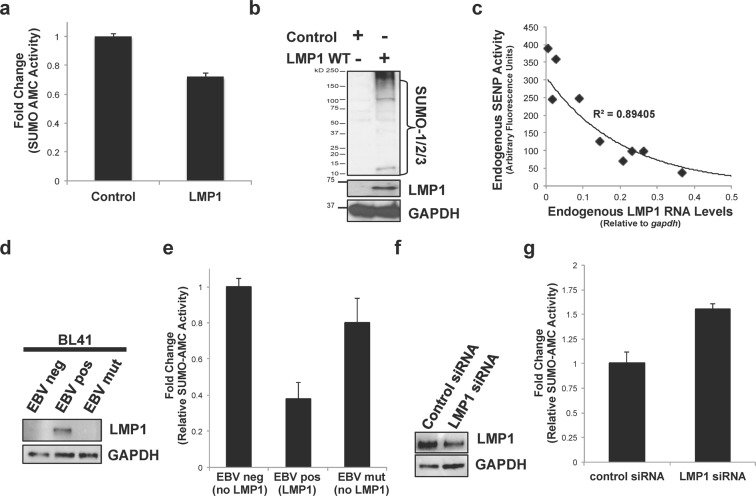
LMP1 expression decreased SENP activity in a dose-dependent manner. (**a**,**b**) HEK 293 cells were transfected as indicated. 48 hours post-transfection, cells were collected, (**a**) SUMO-AMC assays were performed to examine cellular SUMO-protease activity, and (**b**) Western blot analyses were performed to detect levels of endogenously sumoylated proteins and LMP1. GAPDH was used as a loading control. (**c**) Multiple lymphoblastoid cell lines were grown and cells collected. SUMO-AMC assays were performed to examine endogenous SENP activity (represented in arbitrary fluorescence units) and real-time PCR for PCR to quantitate endogenous LMP1 RNA levels (relative to *gapdh*). Regression analysis was performed. (**d**) Western blot analyses on the paired BL41 cell lines were performed to detect LMP1 and GAPDH levels. (**e**) SUMO-AMC assays were performed on lysates from paired BL41 cell lines (No EBV - lack EBV and do not express LMP1; EBV WT - EBV-infected and express LMP1; P3HR1 - infected with a mutant EBV that lacks EBNA2 resulting in no detectable LMP1 expression). Results are shown as mean ± standard deviation of samples done in triplicate and experiments performed in triplicate. (**f**) Western blot analyses were performed using lysates from transfected Raji cells to detect LMP1 and GAPDH. (**g**) SUMO-AMC assays were performed on lysates from Raji cells transfected with LMP1-specific or control siRNAs. Results are shown as mean ± standard deviation of samples done in triplicate and experiments performed in triplicate.

Previously, we documented that LMP1 hijacks Ubc9 using CTAR3 and increases the global sumoylation of cellular proteins^15^. Our recent work established that LMP1-mediated signaling through CTAR1 and CTAR2 induced the *sumo* promoters, increasing intracellular SUMO levels^41^. Together, these events contribute to the observed increased protein sumoylation in LMP1-positive lymphomas^41^, which prompted us to question if LMP1 could modulate other steps of the sumoylation process. Because the SUMO-proteases are required for SUMO maturation and protein de-sumoylation^33^, we first investigated if LMP1 affected SENP activity by quantitating the ability of the endogenous SENPs to cleave a SUMO-modified substrate in LMP1- and control-expressing HEK 293 cells. Findings showed that SUMO-protease activity was significantly (P < 0.05) decreased in LMP1-expressing cells when compared with control-expressing cells (Fig. 1a). In LMP1-expressing cells, the decreased SENP activity corresponded with a detectable increase of SUMO-1/2/3 levels (Fig. 1b and Supplementary Data), confirming our earlier reports that LMP1 expression coincided with increased sumoylation during EBV latency^15,27,32,40^ and suggesting that inhibition of SENP2 activity may augment the accumulation of sumoylated proteins detected in LMP1-positive lymphomas.

Endogenous SUMO-protease activity was quantitated in multiple EBV-transformed lymphoblastoid cell lines, which expressed varying levels of LMP1 RNA (Fig. 1c). Results showed that as LMP1 RNA levels increased, endogenous SUMO-protease activity decreased, almost exponentially, and the data suggested a negative correlation between SUMO-protease activity and LMP1 levels.

To elucidate if LMP1 expression was sufficient to inhibit SUMO-protease activity, the cleavage of SUMO-AMC was analyzed in a set of paired cell lines^42–44^. No EBV (BL41 EBV negative cells) are B-cells that are not infected with EBV so they fail to express LMP1. EBV WT (BL41 EBV WT) are B-cells that are infected with wild-type EBV and express detectable levels of LMP1. P3HR1 (BL41 EBV mutant) are B-cells infected with a mutant EBV where EBNA2 is deleted, which results in no detectable LMP1 expression (Fig. 1d). Analysis of SENP activity in the paired cell lines showed that EBV WT cells had significantly (P < 0.05) lower SUMO-protease activity than their non-LMP1-expressing counterparts (Fig. 1e).

The effect of LMP1 on SENP activity was confirmed using knock-down of LMP1 levels in Raji cells by siRNA (Fig. 1f). Results showed that knock-down of LMP1 resulted in a significant (P < 0.05) increase in SUMO-protease activity when compared with their control siRNA-transfected counterparts (Fig. 1g). Together, these data suggest that LMP1 inhibits SENP2 activity, which led us to hypothesize that, in addition to activating the *sumo* promoters and hijacking Ubc9, LMP1 inhibits SENPs function resulting in increased levels of sumoylated proteins observed in LMP1-expressing cells.

### LMP1 expression induced the sumoylation of SENP2

**Figure 2:**
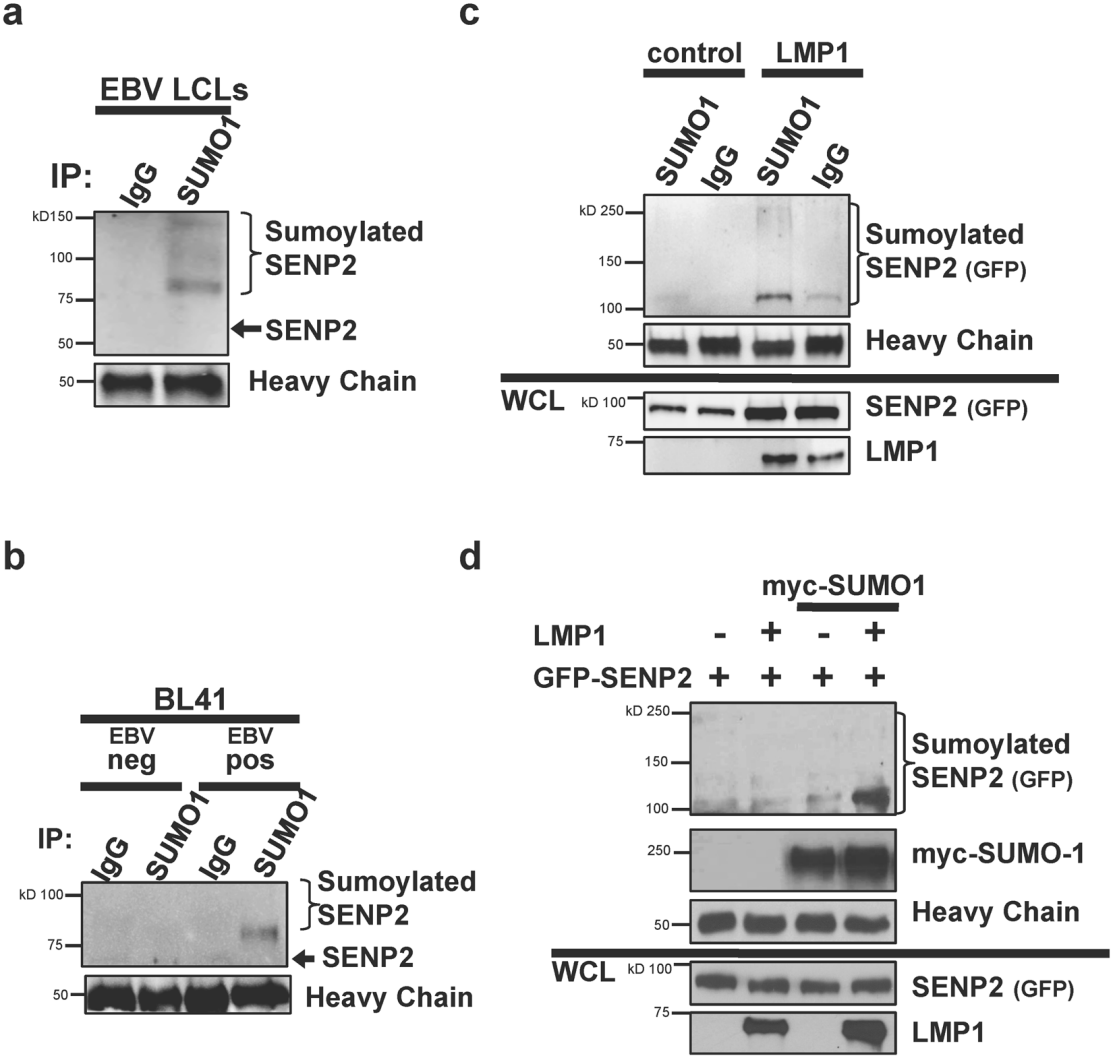
LMP1 induced the sumoylation of SENP2. (**a**) EBV-transformed LCLs were grown and denaturing immunoprecipitations (to pull-down all proteins covalently modified by SUMO-1) were done. (**b**) Denaturing immunoprecipitations were performed on lysates from paired EBV-negative and EBV-positive BL41 B-cells. (**c**,**d**) HEK 293 cells were transfected and denaturing immunoprecipitations were performed using antibodies specific to (**c**) endogenous SUMO-1 or its IgG isotype control or (**d**) exogenous myc-SUMO1. Western blot analyses were done to detect SENP2 (GFP), LMP1, and/or myc-SUMO1 levels.

To begin to understand the effect of LMP1 on the SENPs, we focused on SENP2. We previously documented that LMP1 increased the sumoylation of two specific target proteins (IRF7 and KAP1), regulating their function^27,32^, so we were curious if LMP1 induced the sumoylation of SENP2. To pull-down all proteins covalently modified by endogenous SUMO-1, denaturing immunoprecipitations of EBV-transformed lymphoblastoid cell lines (LCLs) were performed to determine if SENP2 was sumoylated endogenously during EBV latency (Fig. 2a). SENP2 is approximately 68 kDa in size, and results showed that slower migrating forms of SENP2 were pulled-down with anti-SUMO-1 antibodies but not the IgG-isotype control antibodies. These slower migrating forms of SENP2 were recognized by SUMO1-specific antibodies (for the immunoprecipitation) and SENP2-specific antibodies (by Western Blot analyses). In addition, the lowest slower migrating band runs at approximately 12 kDa slower than un-modified SENP2. Therefore, we proposed that the detected bands correspond with endogenously sumoylated SENP2.

To determine if the detection of sumoylated SENP2 was dependent on the presence of EBV, we analyzed EBV-negative and EBV-positive paired BL41 cell lines (Fig. 2b)^42–44^. Denaturing immunoprecipitations revealed that endogenous levels of sumoylated SENP2 were observed in the EBV-positive B-cells but not their EBV-negative counterparts or the control immunoprecipitations, which suggests that SENP2 is endogenously sumoylated during latent EBV infections.

Because we identified a role for LMP1 in regulating sumoylation processes during latent EBV infections^15,27,32,45^, we next investigated if LMP1 was sufficient to induce the sumoylation of SENP2. First, HEK 293 cells were transfected with control- or LMP1-expression constructs. 48 hours post-transfection, lysates were divided and denaturing immunoprecipitations performed using SUMO1-specific antibodies or IgG isotype control antibodies (Fig. 2c). Sumoylated SENP2 was readily detectable in LMP1-expressing cells. Levels of sumoylated SENP2 were almost undetectable in control-expressing cells or the control immunoprecipitations, which suggests that LMP1 is sufficient to induce the sumoylation of SENP2.

Confirming these results, similar experiments were performed with the exception that half of the samples expressed exogenous myc-SUMO-1. To pull-down all proteins covalently modified by myc-SUMO-1, denaturing immunoprecipitations were performed using myc-specific antibodies. Immunoprecipitations performed on the lysates from cells not expressing myc-SUMO-1 served as experimental controls, and as expected, sumoylated SENP2 was not detected in either control sample. Furthermore, sumoylated SENP2 (modified by exogenous myc- SUMO-1) was only detected when LMP1 was co-expressed (Fig. 2d). Together, these results led us to propose that LMP1 induces the sumoylation of SENP2.

### LMP1 expression coincided with decreased SENP2 function

**Figure 3:**
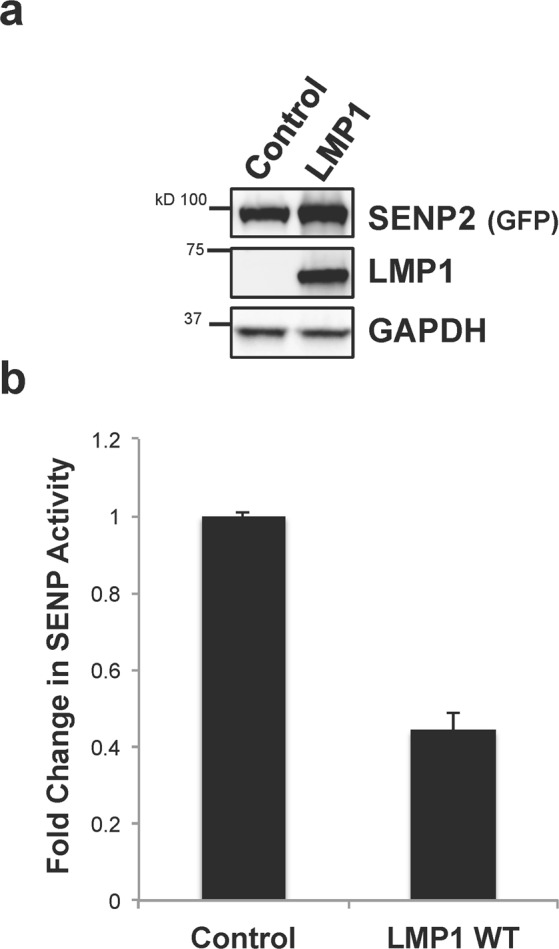
LMP1 expression coincided with decreased SENP2 function. HEK 293 cells were transfected as indicated along with a SENP2-expression vector. 48 hours post-transfection, cells were collected. (**a**) Western blot analyses of SUMO-AMC lysates were performed to determine SENP2 (GFP), LMP1, and GAPDH levels. (**b**) SUMO-AMC assays were performed to examine SENP activity. Results are shown as mean ± standard deviation of samples done in triplicate and experiments performed in triplicate.

Protein sumoylation can affect a protein’s biology in a multitude of manners^1–3,46,47^. Because we detected decreased SENP activity in LMP1-expressing cells, we next determined the effect of LMP1 on SENP2 function. Cells were transfected with SENP2 and control- or LMP1-expression constructs. Transfected cell lysates (Fig. 3a) were used to quantitate changes in SENP activity, which were normalized to overall SENP2 levels (Fig. 3b). Results showed that lysates co-expressing LMP1 and SENP2 had a significantly (P < 0.05) decreased ability to process a sumoylated substrate than lysates from control-expressing cells, which suggests that SENP2 activity is inhibited in the presence of LMP1.

### LMP1 expression increased the stability of SENP2

**Figure 4:**
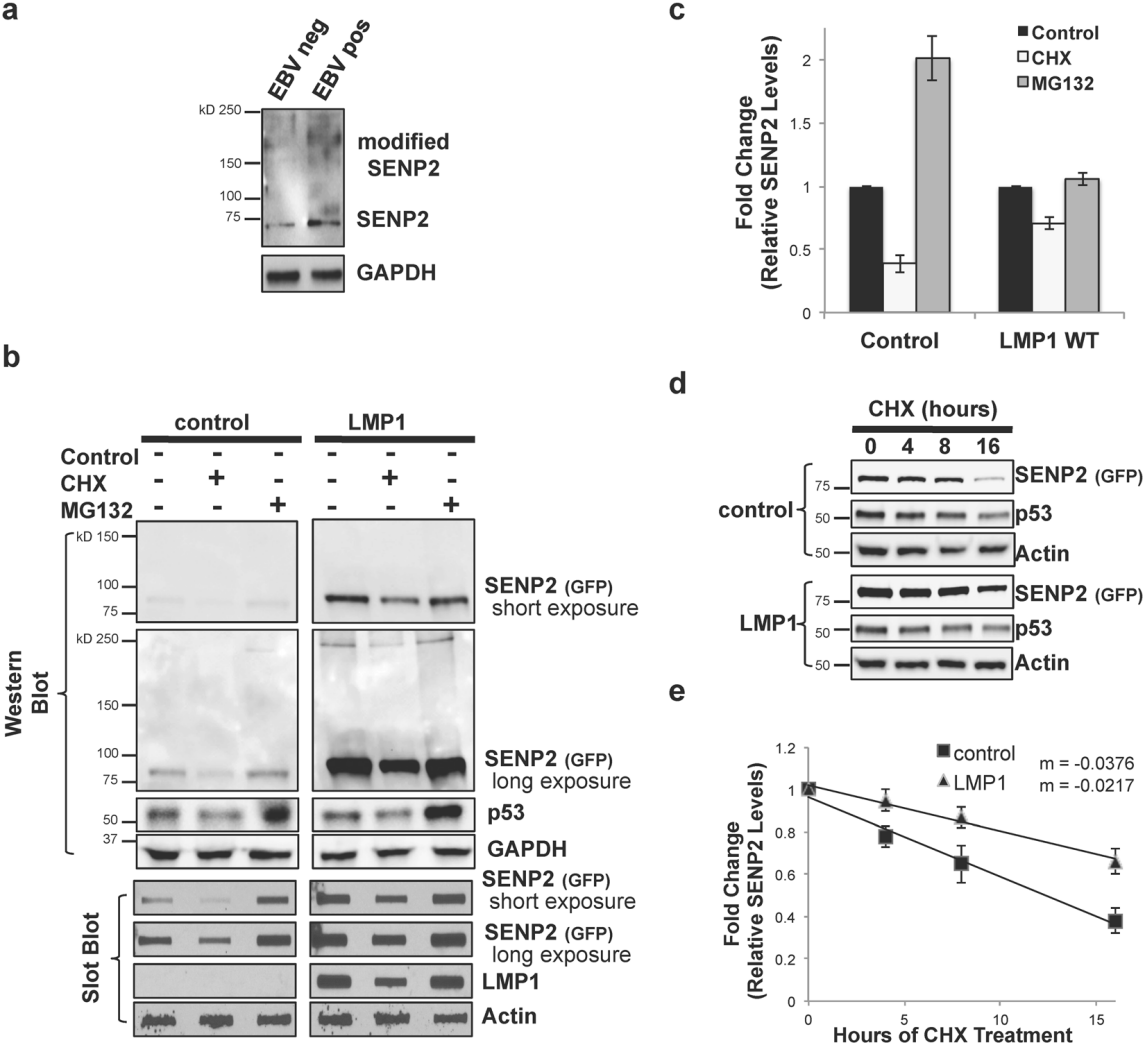
LMP1 expression increased SENP2 stability. (**a**) Western blot analyses were performed on lysates from paired EBV-negative and EBV-positive BL41 B-cells to detect endogenous SENP2 levels. GAPDH was used as a loading control. (**b**,**c**) HEK 293 cells were transfected as indicated and treated with cyclohexamide (CHX), MG132, or the vehicle control (DMSO) for 18 hours prior to harvesting. (**b**) Western blots and slot blot analyses were performed to detect total SENP2 levels. (**c**) Densitometric analysis was performed on repeat experiments. Results are shown as mean ± standard deviation of experiments performed in triplicate. (**d**,**e**) HEK 293 cells were transfected with control- or LMP1-expression constructs, and treated with cyclohexamide for 0, 4, 8, or 16 hours prior to harvesting. (**d**) Western blot analyses were performed to detect total endogenous SENP2 levels. Actin was used as a loading control. (**e**) Densitometric analysis was performed on repeat experiments. Results are shown as mean ± standard deviation of experiments performed in triplicate.

In addition to regulating the function of a protein, sumoylation can compete with ubiquitination, specifically K48-linked ubiquitination, to increase the protein’s stability and decreasing its turnover. In earlier experiments, LMP1-expressing cells had higher endogenous SENP2 levels when compared with control-expressing cells, so we examined endogenous SENP2 levels in EBV-negative and EBV-positive BL41 cell lines (Fig. 4a)^42–44^. Results showed that EBV-positive B-cells had increased SENP2 levels when compared with EBV-negative B-cells. In addition, increased slower-migrating, modified SENP2 was only detected in the EBV-positive cells. The findings led us to propose that the stability of SENP2 may be affected during latent EBV infections.

To examine the turnover of SENP2, HEK 293 cells were transfected and treated with DMSO, cyclohexamide (CHX), or a proteasome inhibitor (MG132) for 18 hours. Prior to harvesting, cell survival was determined and no changes in cell survival were detected (data not shown). Relative total SENP2 levels were determined for Western blot and slot blot analyses, which specifically allows for detection of modified and un-modified SENP2 levels (Fig. 4b,c)^48^. p53 was used as an experimental control while GAPDH or Actin were used as loading controls. In control-expressing cells CHX treatment resulted in a significant (P < 0.05) decrease in SENP2 levels while MG132 treatment increased SENP2 levels. These findings suggest that SENP2 is normally targeted for degradation by the proteasome with a significant amount of turnover detected by 18 hours post-treatment. Expression of LMP1 coincided with significantly (P < 0.05) less of a decrease in SENP2 levels following cyclohexamide treatment and treatment with MG132 did not result in the accumulation of SENP2. These data indicate that expression of LMP1 increased the stability of SENP2 and inhibited the targeting of SENP2 to the proteasome.

The effect of LMP1 on the stability of SENP2 was further examined over a course of cyclohexamide treatment (Fig. 4d,e). In both control- and LMP-expressing cells, SENP2 levels decreased as hours of cyclohexamide treatment increased. Comparison of the rate of loss of SENP2 revealed that SENP2 levels decreased at a higher rate in control-expressing cells when compared with LMP1-expressing cells. These data provide further evidence that expression of LMP1 stabilizes SENP2 levels.

### LMP1 expression affected SENP2 localization and the accumulation of SENP2 at the nuclear envelope

**Figure 5:**
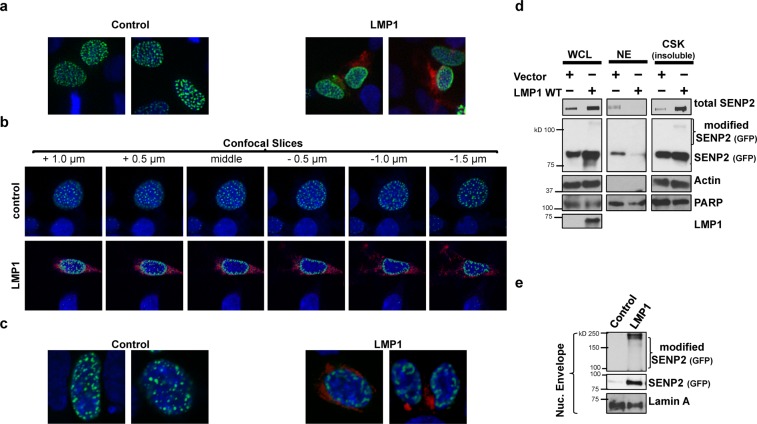
LMP1 expression affected SENP2 localization and the accumulation of SENP2 in the nuclear envelope. HEK 293 cells were transfected as indicated along with a GFP-SENP2-expression vector. (**a**–**c**) 24 hours post-transfection, cells were fixed, permeabilized, and stained for LMP1 and DAPI. (**a**) Immunofluorescence microscopy was performed at 20X magnification using the EVOS FLoid Cell Imaging Station. (**b**,**c)** Confocal microscopy was performed at 60X magnification using the Nikon A1 laser confocal microscope. (**b**) Z-stacked images were taken every 0.5 μm. (**c)** Representative images of the middle slice of the nucleus are shown. (**d**,**e)** HEK 293 cells were transfected as indicated. 48 hours post-transfection (**d**) Whole cell lysates (WCL) and nuclear extracts (NE), and cytoskeletal fractions (CSK) were collected and (**e**) nuclear envelopes were isolated. Western blot analyses to detect SENP2 (GFP) and LMP1 were performed, and PARP, Actin, and Lamin A were used as loading controls.

In addition to regulating protein function and turnover, protein sumoylation can modulate a protein’s trafficking and localization. Immunofluorescence microscopy was performed to examine the localization of SENP2 within the cell. SENP2 normally localizes to the nuclear side of the nuclear pore complex^49^, and punctate SENP2 staining was observed, suggestive of SENP2 localizing at the nuclear pores (Fig. 5a). When LMP1 was co-expressed, SENP2 accumulated at the periphery of the nucleus. Confocal microscopy was performed to confirm that LMP1 expression altered the localization of SENP2 (Fig. 5b,c). Z-stacks (0.5 μm slices) images of nuclei were taken (representative stack Fig. 5b). In control cells, SENP2 localizes throughout the nucleus (center and with small amounts at the edges of the nucleus). Marked differences were observed when observing SENP2 localization in LMP1-expressing cells. Specifically, the majority of SENP2 was detected at the periphery of the nucleus. Comparison of images collected at the middle of nuclei confirmed the consistent movement of SENP2 from the middle of the nuclei to the periphery of the nuclei when LMP1 was expressed (Fig. 5c). These results demonstrate that LMP1 expression does alter SENP2 localization within the nuclei.

To gain understanding into the changes occurring in the localization of SENP2, different cellular compartments were isolated (Fig. 5d,e). While SENP2 normally localized with the nuclear extracts (NE) and cytoskeletal/insoluble proteins (Fig. 5d), expression of LMP1 resulted in loss of SENP2 localization to the nuclear extracts and accumulation of SENP2 with the cytoskeletal/insoluble proteins. Furthermore, isolation of the nuclear envelope from the cytoskeletal/insoluble protein extracts revealed that LMP1 expression specifically resulted in the accumulation of SENP2 (and modified SENP2) in the nuclear envelope when compared with control-expressing cells (Fig. 5e). These findings suggest that LMP1 alters SENP2 localization by removing it from the nuclear side of the nuclear pore and increasing its accumulation within the nuclear envelope. Together, the data led us to propose that LMP1 induces the sumoylation of SENP2, regulating SENP2 function, stability, and trafficking. Therefore, next we wanted to identify the requirements for LMP1-mediated sumoylation of SENP2.

### LMP1-mediated induced the sumoylation of SENP2 in a CTAR-dependent manner

**Figure 6:**
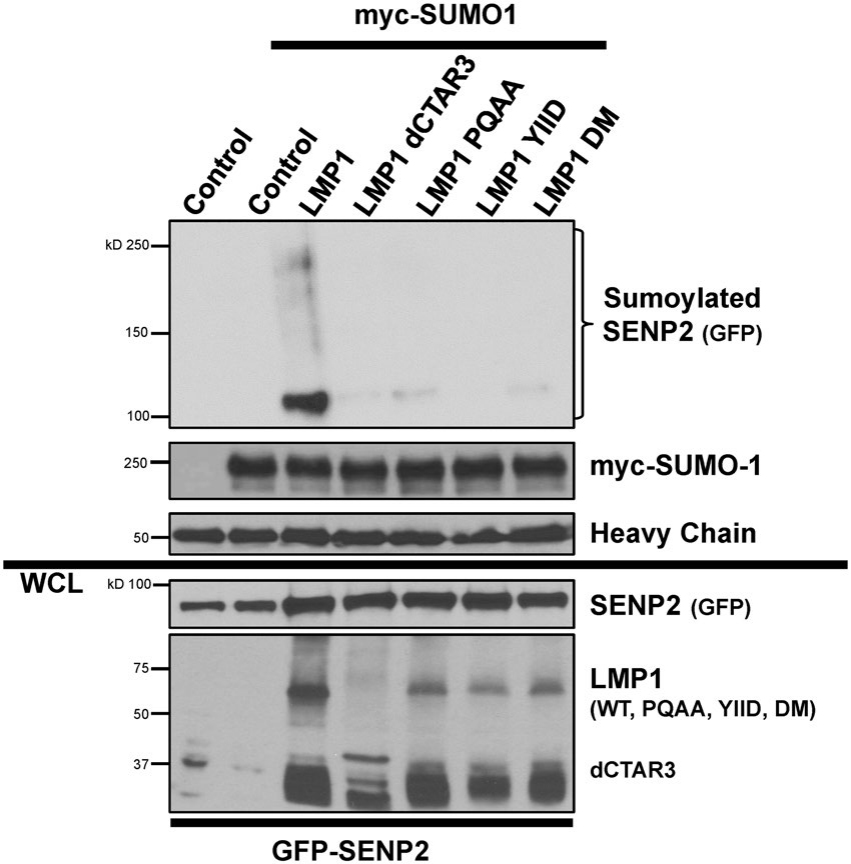
LMP1-induced the sumoylation of SENP2 in a CTAR-dependent manner. HEK 293 cells were transfected as indicated. 48 hours post-transfection, cells were collected. Denaturing immunoprecipitations were performed to pull-down all proteins covalently modified by myc-SUMO1. Western blot analyses were done to detect SENP2 (GFP), LMP1, and myc-SUMO1 levels.

To begin to decipher the requirements of LMP1-induced sumoylation of SENP2, we first focused on the LMP1 requirements for SENP2 sumoylation. Using select LMP1 mutants that are lacking functional CTAR1, CTAR2, and/or CTAR3 domains, we examined the LMP1 regulatory region required for the observed sumoylation of SENP2 (Fig. 6). Denaturing immunoprecipitations were performed to pull-down all proteins covalently modified by exogenous myc-SUMO1, with non-myc-SUMO-1-transfected cells serving as the experimental controls. Results confirmed that sumoylated SENP2 was detected when LMP1 was expressed, and not in control-expressing cells or the experimental control. Deletion of CTAR3 (LMP1 dCTAR3; lacking amino acids 250–307), mutation of CTAR1 (LMP1 PQAA) and/or CTAR2 (LMP1 YIID and LMP1 DM, in which both CTAR1 and CTAR2 are mutated) resulted in decreased detection of sumoylated SENP2 (Fig. 6), which suggests that all three C-terminal activating regions are necessary for LMP1-induced sumoylation of SENP2.

### LMP1 modulated SENP2 function, turnover, and localization in a CTAR-dependent manner

**Figure 7:**
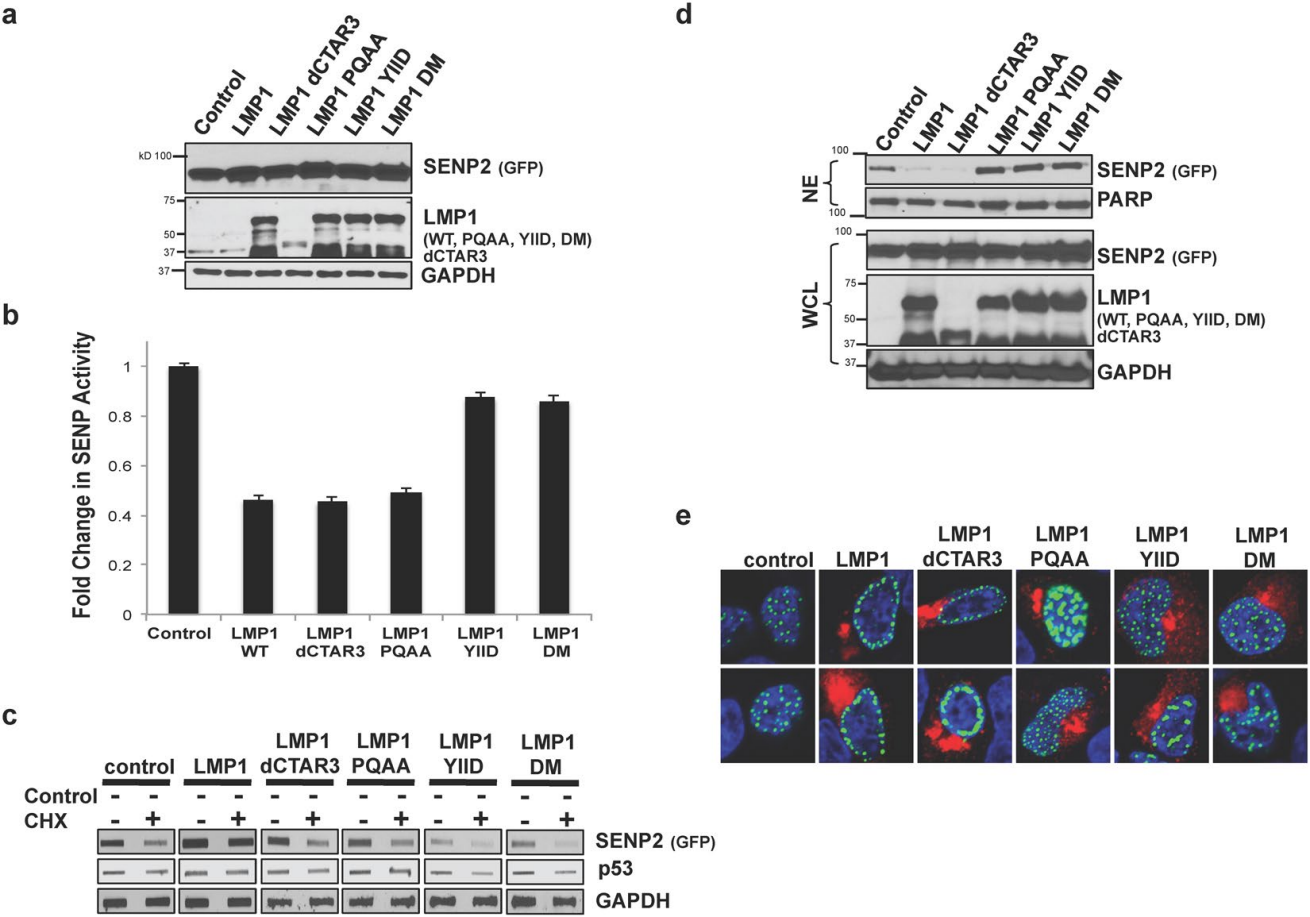
LMP1 modulated SENP2 function, turnover, and localization in a CTAR-dependent manner. HEK 293 cells were transfected as indicated. **(a**,**b)** 48 hours post-transfection, cells were collected. (**a**) Western blot analyses of SUMO-AMC lysates were performed to determine SENP2 (GFP), LMP1, and GAPDH levels. (**b**) SUMO-AMC assays were performed, and results are shown as mean ± standard deviation of samples done in triplicate and experiments performed in triplicate. (**c**) Cells were treated with cyclohexamide (CHX) or the vehicle control (DMSO) for 18 hours prior to harvesting. Slot blot analyses were performed to detect total SENP2 levels. (**d**) WCL and NE fractions were collected. Western blot analyses were performed to detect SENP2 (GFP) and LMP1. GAPDH and PARP were used as loading controls. (**e**) Confocal microscopy was performed at 60X magnification using the Nikon A1 laser confocal microscope. Representative images of the middle slice of the nucleus are shown.

Next, we identified the LMP1 regulatory region(s) involved in LMP1-mediated modulation of SENP2 function, stability, and trafficking. First, the effect of the select LMP1 mutants on SENP2 function was quantitated (Fig. 7a,b). Data again confirmed that expression of LMP1 corresponded with a significant (P < 0.05) decrease in SENP levels. Loss of CTAR1 or CTAR3 did not affect LMP1-mediated inhibition of SENP activity. However, loss of a functional CTAR2 domain almost completely reversed the effect of LMP1 on SENP activity (Fig. 7b), suggesting that only LMP1 CTAR2 was necessary for LMP1-mediated inhibition of SENP activity.

The select LMP1 mutants were also used to determine the requirements for LMP1-mediated regulation of SENP2 turnover. Results from control-transfected cells confirmed that 18 hours of cyclohexamide treatment resulted in a significant decrease in SENP2 levels (Fig. 7c). Confirming our earlier findings, LMP1 expression corresponded with less SENP2 turnover. Mutation of any of the LMP1 CTARs abolished LMP1-induced stabilization of SENP2 levels, which suggests that all three CTARs cooperate in LMP1-mediated increased stability of SENP2.

To examine the requirements for LMP1-mediated regulation of SENP2 localization, different cellular compartments were harvested from transfected cells (Fig. 7d). Comparison of control- and LMP1-expressing cells confirmed that LMP1 expression resulted in the altered localization of SENP2, with the accumulation of SENP2 in the insoluble fraction with cytoskeletal proteins (Fig. 7d). Mutation of either CTAR1 or CTAR2 restored SENP2 localization to the nuclear extracts (Fig. 7d), with loss of CTAR3 having no effect on LMP1-mediated altered SENP2 localization. SENP2 localization was also examined by confocal microscopy (Fig. 7e), which confirmed that LMP1 expression coincided with the accumulation of SENP2 at the periphery of the nucleus while control-expressing cells had SENP2 throughout the nucleus. Deletion of CTAR3 did not change the effect of LMP1 on SENP2 localization. However, mutation of CTAR1 and/or CTAR2 abrogated the ability of LMP1 to alter the localization of SENP2, which suggests that CTAR1 and CTAR2 are required for LMP1-induced alteration of SENP2 localization.

These findings confirm that LMP1 regulates SENP2 function, turnover, and localization. While all three CTARs seem to cooperate in LMP1-mediated modulation of SENP2 stability and localization, CTAR2 is the main regulatory domain involved in inhibiting SENP2 function. In addition, the finding that all three CTARs were not required for LMP1-induced modulation of SENP2 biology, alludes to the idea that LMP1-induced sumoylation of SENP2 was not necessary for the observed changes in SENP2 biology.

### LMP1 induced the sumoylation of SENP2 at K48 and K447

**Figure 8:**
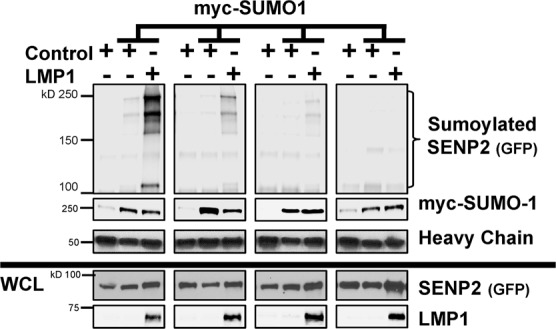
LMP1 induced the sumoylation of SENP2 at K48 and K447. HEK 293 cells were transfected as indicated. 48 hours post-transfection, cells were collected and denaturing immunoprecipitations were performed to pull-down all proteins covalently modified by myc-SUMO1. Western blot analyses were done to detect SENP2 (GFP), LMP1, and myc-SUMO1 levels.

Although we deduced that LMP1-induced sumoylation of SENP2 was not the mechanism by which LMP1 modulated the biology of SENP2, we still wanted to identify the SENP2 residues modified by sumoylation in the presence of LMP1. Analysis of the SENP2 sequence by SUMOplot^TM^ (Abgent) revealed three probable conserved sumoylation motifs (K48, K118, and K447). Of these lysine residues, SENP2 K447 is in direct contact with SUMO-1 during the de-sumoylation process^34^ and SENP2 K48 resides within the nuclear localization sequence^50^. Therefore the function of these two lysine residues in LMP1-mediated regulation of SENP2 function was determined. Site directed mutagenesis, conserving the charge of the lysine residue, was performed to yield three mutants (K48R, K447R, and K48R/K447R; Fig. 8). Denaturing immunoprecipitations for proteins covalently modified by exogenous myc-SUMO1 confirmed that LMP1 expression coincided with increased levels of sumoylated SENP2. myc-SUMO1-modified SENP2 was not detected in control-expressing cells or in the experimental control (cells lacking exogenous myc-SUMO1). Mutation of SENP2 K48 or K447 significantly decreased levels of detectable sumoylated SENP2 in LMP1-expressing cells. LMP1-induced sumoylation of SENP2 was completely abrogated when both SENP2 K48 and K447 were mutated, which suggests that LMP1 induces the sumoylation of these two lysine residues.

### LMP1-induced modulation of SENP2 activity was dependent on SENP2 K447

**Figure 9:**
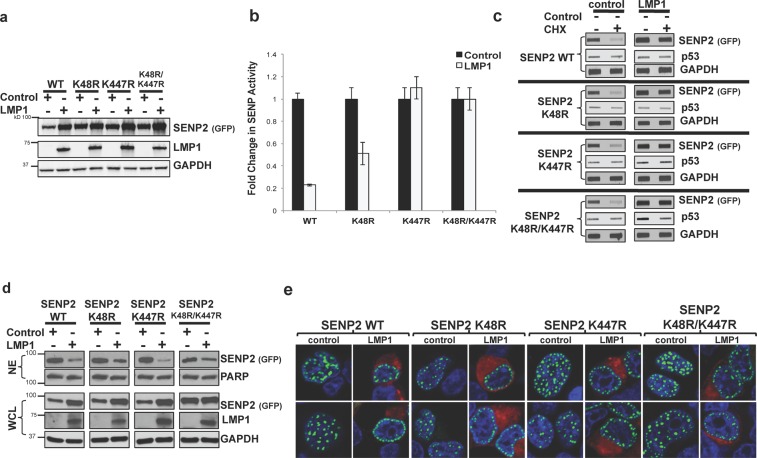
LMP1-induced sumoylation of SENP2 at K48 and K447 did not contribute to LMP1-mediated modulation of SENP2 stability and localization. HEK 293 cells were transfected as indicated. 48 hours post-transfection, cells were collected and (**a**) Western blot analyses of SUMO-AMC lysates were performed to determine SENP2 (GFP), LMP1, and GAPDH levels. (**b**) SUMO-AMC assays were performed, and results are shown as mean ± standard deviation of samples done in triplicate and experiments performed in triplicate. (**c**) treated with cyclohexamide (CHX) or the vehicle control (DMSO) for 18 hours prior to harvesting. Slot blot analyses were performed to detect total SENP2 levels. (**d**) WCL and NE fractions were collected. Western blot analyses were performed to detect SENP2 (GFP) and LMP1. GAPDH and PARP were used as loading controls. (**e**) Confocal microscopy was performed at 60X magnification using the Nikon A1 laser confocal microscope. Representative images of the middle slice of the nucleus are shown.

While our findings suggested that LMP1-induced sumoylation of SENP2 was not necessary for the observed changes in SENP2 biology, we still wanted to identify if the SENP2 sumoylation sites were involved in LMP1-induced modulation of SENP2 function. Analysis of SENP activity revealed that that mutation of SENP2 K48 and/or K447 did not affect SENP activity in control-expressing cells (Fig. 9a,b). Interestingly, mutation of SENP2 K447 reversed LMP1-mediated inhibition of SENP2 activity, while mutation of SENP2 K48 only partially restored LMP1-mediated inhibition of SENP2 activity. These findings further verified that LMP1-induced sumoylation of SENP2 is not involved in LMP1-mediated modulation of SENP2 function but SENP2 K447 is involved in LMP1-induced regulation of SENP2 activity.

### LMP1-induced sumoylation of SENP2 at K48 and K447 did not contribute to LMP1-mediated modulation of SENP2 stability and localization

To better elucidate the role of SENP2 K48 and K447 in LMP1-induced changes in SENP2 biology, the function of these residues in SENP2 stability and localization was examined. Control- and LMP1-expressing cells were treated with cyclohexamide or the vehicle control for 18 hours prior to harvesting. Results showed that mutation of SENP2 K48 and/or SENP2 K447 did not alter SENP2 turnover in control-expressing cells (Fig. 9c). Expression of LMP1 corresponded with increased stability of SENP2 (wild-type and mutants), demonstrating that neither SENP2 K48 nor SENP2 K447 were targets of LMP1-mediated regulation of SENP2 turnover. Together, these results suggest SENP2 K48 and K447 are not required for the normal turnover of the SUMO protease and LMP1 does not increase the stability of SENP2 by targeting either of these residues.

Next the role of SENP2 K48 or SENP2 K447 in LMP1-mediated regulation of SENP2 localization was examined. In control-expressing cells, mutation of SENP2 K48 and/or K447 did not alter the normal nuclear distribution of SENP2 (Fig. 9d,e). Mutation of SENP2 K48 or K447 also did not abrogate LMP1-induced accumulation of SENP2 at the periphery of the nucleus or the loss of SENP2 in nuclear extracts (Fig. 9d,e), which demonstrates that neither lysine residue is targeted by LMP1 for the regulation of SENP2 localization within the cells and further supports the idea that LMP1-induced sumoylation of SENP2 does not regulate the localization of SENP2.

Together, these data demonstrate that LMP1 manipulates SENP2 function, turnover, and localization. However, LMP1-induced sumoylation of SENP2 and SENP2 K48 and K447 are not involved in LMP1-mediated manipulation of the biology of SENP2.

### LMP1 regulated the ubiquitination of SENP2

**Figure 10:**
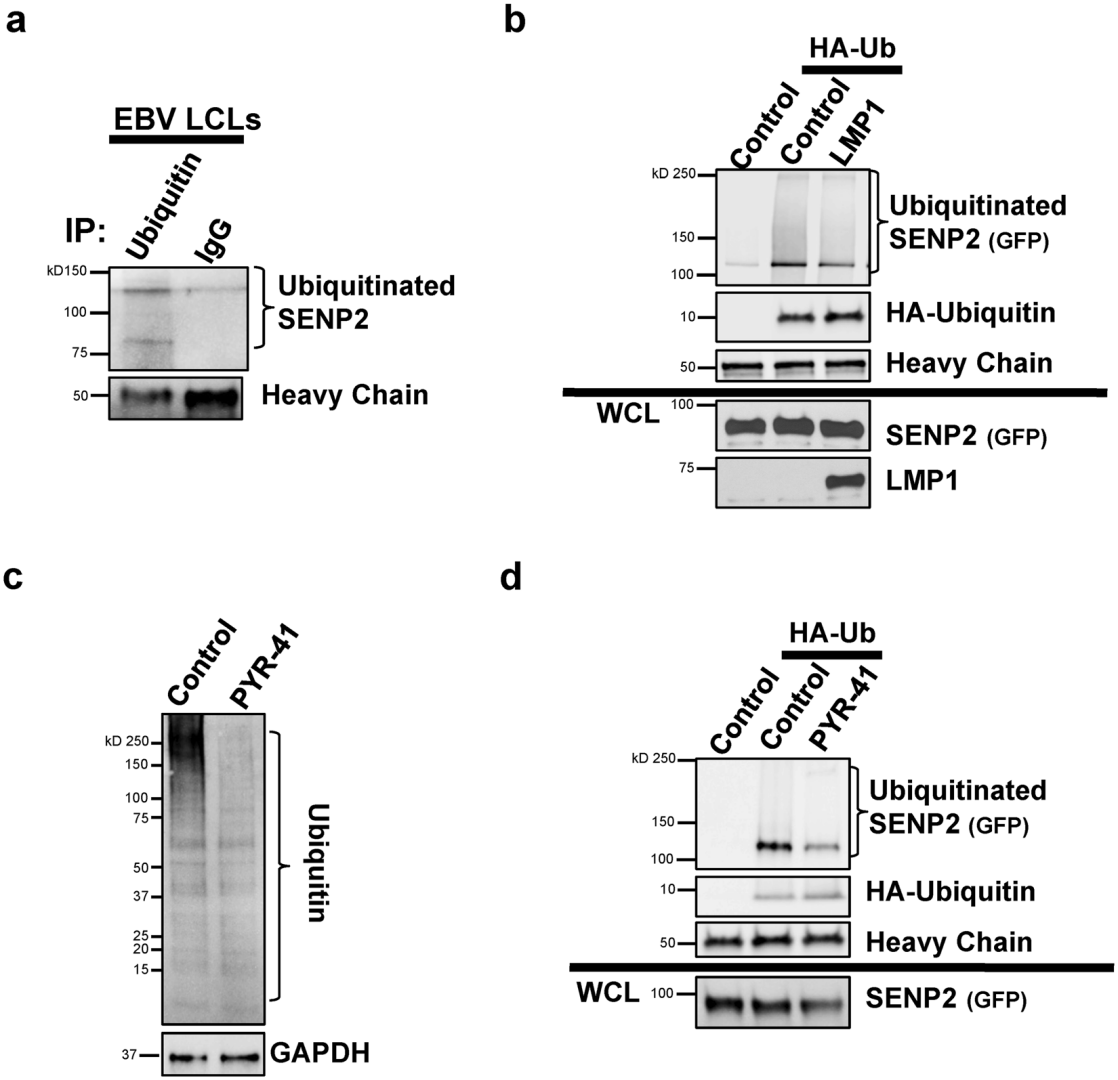
LMP1 regulated the ubiquitination of SENP2. (**a**) EBV-transformed LCLs were grown and denaturing immunoprecipitations (to pull-down all proteins covalently modified by endogenous ubiquitin) were performed. (**b**) HEK 293 cells were transfected and denaturing immunoprecipitations were performed to pull-down all proteins covalently modified by exogenous HA-ubiquitin (HA-Ub). Western blot analyses were done to detect SENP2 (endogenous or GFP), LMP1, and/or myc-SUMO1 levels. (**c**,**d**) HEK 293 cells were transfected as indicated and treated with PYR-41 or the vehicle control (DMSO) for 18 hours prior to harvesting. (**c**) WCL were collected and Western blot analyses performed to detect endogenous ubiquitin levels. GAPDH was used as the loading control. (**d**) Denaturing immunoprecipitations were performed to pull-down all proteins covalently modified by exogenous HA-ubiquitin (HA-Ub). Western blot analyses were performed to detect SENP2 or HA-ubiquitin.

Because we discovered that LMP1-mediated sumoylation of SENP2 was not involved in modulation of SENP2 biology, we wanted to better decipher the mechanism by which the viral oncoprotein affected this cellular protein. SUMO can directly and indirectly compete with ubiquitin during protein post-translation modifications^1,2,51^, so we examined if SENP2 was ubiquitinated during EBV latency. Denaturing immunoprecipitations to pull-down all proteins covalently modified by ubiquitin revealed that slower migrating SENP2 was detected in EBV LCLs when ubiquitin-specific antibodies were used for the immunoprecipitations (Fig. 10a). Similar bands were not detected when immunoprecipitations were performed using IgG isotype control antibodies, leading us to propose that we were detecting ubiquitinated SENP2.

To confirm the role of LMP1 in the ubiquitination of SENP2, denaturing immunoprecipitations were perform on control- and LMP1-expressing lysates, where exogenous HA-ubiquitin was also expressed (Fig. 10b). Immunoprecipitations performed on the lysates from cells not expressing HA-ubiquitin served as the experimental control. In control-expressing cells, a slower migrating form of SENP2 was detected only in cells also expressing exogenous HA-ubiquitin, which suggests we were detecting ubiquitinated SENP2. Surprisingly, lower levels of ubiquitinated SENP2 were detected in LMP1-expressing lysates when compared with control-expressing lysates, which indicated that expression of LMP1 inhibited the ubiquitination of SENP2.

### Inhibition of ubiquitination processes mediated the observed changes in SENP2 biology

To decipher if LMP1-mediated inhibition of SENP2 ubiquitination could account for the observed changes in SENP2 biology, we utilized a known inhibitor of ubiquitin-activating enzymes, PYR-41^52^, which decreased levels of ubiquitinated proteins in treated cells (Fig. 10c). Denaturing immunoprecipitations to detect proteins covalently modified by exogenous HA-ubiquitin were performed on control-expressing cells following treatment with PYR-41 or the vehicle control (Fig. 10d). Cells lacking exogenous HA-ubiquitin were used as the experimental control. Findings showed that HA-ubiquitinated SENP2 was detected in control-treated cells. Substantially lower levels of HA-ubiquitinated SENP2 were detected in cells treated with PYR-41, demonstrating that treatment of PYR-41 inhibited the ubiquitination of SENP2.

**Figure 11:**
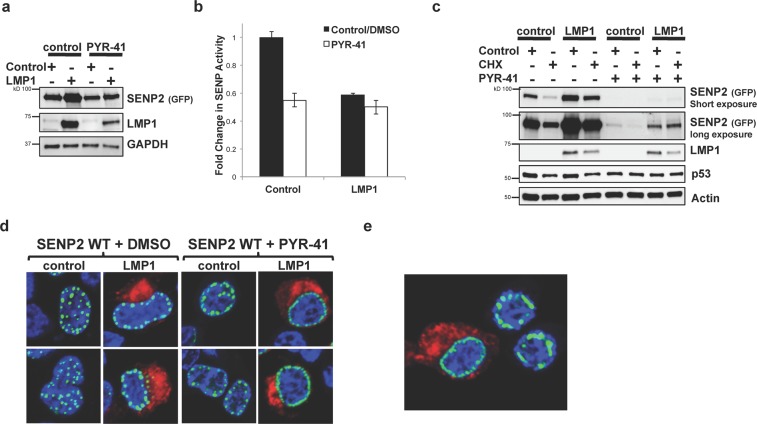
Inhibition of ubiquitination processes mediated the observed changes in SENP2 biology. HEK 293 cells were transfected as indicated and treated with PYR-41 or DMSO for 18 hours prior to harvesting. (**a)** SUMO-AMC lysates were collected and Western blot analyses performed to determine SENP2 (GFP), LMP1, and GAPDH levels. (**b)** SUMO-AMC assays were performed, and results are shown as mean ± standard deviation of samples done in triplicate and experiments performed in triplicate. (**c)** Cells treated with cyclohexamide (CHX) or the vehicle control (DMSO) for 18 hours prior to harvesting. Western blot analyses were performed to detect total SENP2 levels. (**d**,**e**) Confocal microscopy was performed at 60X magnification using the Nikon A1 laser confocal microscope. Representative images of the middle slice of the nucleus are shown. (**e**) Representative image showing LMP1-expressing and non-LMP1-expressing cells that were treated with PYR-41.

First, we quantitated SENP activity in control- and LMP1-expressing cells treated with PYR-41 or its vehicle control (DMSO) (Fig. 11a,b). Confirming our earlier findings, expression of LMP1 coincided with a significant (P < 0.05) decrease in SENP activity when compared with control-expressing cells. Treatment with PYR-41 significantly (P < 0.05) inhibited SENP2 activity in control-expressing cells, but did not affect SENP activity in LMP1-expressing cells, which suggests the mechanism by which LMP1 modulated SENP activity was by inhibiting SENP ubiquitination.

Second, we analyzed the effect of PYR-41 on SENP2 turnover following treatment with cyclohexamide. As before, results showed that LMP1-expression resulted in decreased turnover of SENP2 when compared with control-expressing cells when cells were treated with the vehicle control (Fig. 11c). Treatment of cells with PYR-41 did not affect SENP2 turnover in either control- or LMP1-expressing cells, suggesting that inhibition of ubiquitination processes was not sufficient to increase the stability of SENP2 or affect LMP1-mediated decreased turnover of SENP2.

Third, confocal microscopy of was performed to examine the effect of PYR-41 on SENP2 localization. In control-treated cells (DMSO), expression of LMP1 resulted in the accumulation of SENP2 at the periphery of the nucleus (Fig. 11d). While the localization of SENP2 did not change in LMP1-expressing cells when they were treated with the ubiquitination inhibitor, PYR-41-treatment of control-expressing cells also promoted the relocalization of SENP2 to the periphery of the nucleus. Furthermore, images were captured where the similar peripheral nuclear localization of SENP2 was seen in LMP1-expressing and non-LMP1-expressing cells within the same field of view, which was only detected when cells were treated with PYR-41 (Fig. 11e). These data suggest that LMP1-mediated inhibition of SENP2 ubiquitination promoted the altered SENP2 localization within the cell and the accumulation of SENP2 at the periphery of the nucleus.

Together, the data showed that LMP1 modulates the activity, stability, and trafficking of SENP2. In addition, LMP1 induces the sumoylation of SENP2; however, LMP1-mediated sumoylation of SENP2 is not the mechanism by which LMP1 regulates the biology of SENP2. Instead, LMP1 inhibited the ubiquitination of SENP2, modulating the activity and localization of the SUMO-protease. These findings provide additional evidence that LMP1 modulates sumoylation processes and identify a third manner by which LMP1 manipulates the sumoylation machinery during latent EBV infections. In addition, these results demonstrate the need for an even better understanding of how the viral oncoprotein affects SENP2.

## Discussion

Previously, we documented a new function for LMP1 when we identified that the viral oncoprotein could modulate cellular sumoylation processes during EBV latency by hijacking the SUMO-conjugating enzyme (Ubc9)^15^, which raised the following question; are there any other mechanisms by which LMP1 manipulates the sumoylation machinery? Here, we identify an additional mode by which LMP1 can target sumoylation processes, specifically by dysregulating SUMO protease activity. We specifically focused on the ability of LMP1 to affect SENP2, a member of the first SENP sub-family that cleaves SUMO-1-, -2-, and -3-modified proteins^33^. We showed that LMP1 regulated SENP2 function, turnover, and localization in a CTAR-dependent manner. While LMP1 did induce the sumoylation of SENP2, the viral oncoprotein modulated SENP2 biology independent of SENP2 sumoylation. Finally, here we document that LMP1 inhibited the ubiquitination of SENP2, modulating SENP2 activity and localization. Together with our previous work, these findings demonstrate that LMP1 manipulates multiple steps of the sumoylation process in order to regulate sumoylation events during latent infections.

The finding that EBV-transformed LCLs and LMP1-expressing Burkitt’s lymphoma cells had reduced SENP activity compared with their naïve or LMP1-negative counterparts, led us to investigate how LMP1 regulates SENP function. We focused on SENP2 due to its ability to cleave SUMO-modified proteins^33^. Detecting protease activity in SENP2-over-expressing cells allowed us to semi-quantitate SENP2 activity, and results revealed that expression of LMP1 decreased the ability of SENP2 to cleave SUMO-modified proteins. However, this decrease is not likely relegated to only SENP2. Instead, the decreased LCL SENP activity is most likely due to LMP1 expression inhibiting the function of multiple SENPs.

While sumoylation of SENP1 has been previously reported^53^, the functional consequence of SENP1 sumoylation has remained unknown^53^. Similarly, here we identify that SENP2 is sumoylated, but the functional consequence of SENP2 sumoylation remains unknown. Although we could not deduce the effects of LMP1-mediated sumoylation of SENP2, we still identified the third target of LMP1-induced sumoylation. Our previous work reported that LMP1 mediates the sumoylation of two transcription factors, interferon regulatory factor-7 (IRF7) and KRAB-associated protein-1 (KAP1), which results in their transcriptional repression^27,32^. Here, we identify the first non-transcription factor target of LMP1-induced sumoylation. Sumoylated SENP2 was only detected in LMP1-expressing cells, and our data show that SENP2 is sumoylated in a LMP1 CTAR1-, CTAR2-, and CTAR3-dependent manner at K48 and K447. This work is the first to identify cooperation between all three CTARs in LMP1-induced sumoylation of a cellular protein. Our previous work documented that LMP1 CTAR2 and CTAR3 cooperate in the induction of the sumoylation of IRF7^32^ and that CTAR1 and CTAR2 cooperate to induce *sumo* levels^40^. However, only CTAR3 was required for LMP1-mediated sumoylation of KAP1^27^. The proposed functions for LMP1 CTAR1 and CTAR2 in the manipulation of the sumoylation process were limited to the ability of the two CTARs to activate NF-κB^27^ and the indirect interaction between CTAR2 and IRF7^32^. This is the first work showing cooperation between the three CTARs in the sumoylation of SENP2 and future work will focus on deciphering the specific role of each CTAR in the regulation of SENP2 sumoylation and biology.

While we identified K48 and K447 as the amino acids in SENP2 targeted for sumoylation by LMP1, mutation of these two lysine residues would inhibit sumoylation along with any other lysine-specific post-translational modifications, such as ubiquitination or acetylation. Mutation of K48 and/or K447 did not affect SENP2 activity, stability, or localization in control-expressing cells. These findings suggest that SENP2 K48 and K447 are not required in the normal biology of SENP2 and are not post-translationally modified under normal conditions. In LMP1-expressing cells, mutation of SENP2 K48 and/or K447 did not affect LMP1-induced regulation of SENP2 turnover or localization, implying that these two lysine residues are not critical for LMP1-mediated effects on SENP2 stability and trafficking.

Interestingly, mutation of SENP2 K447 did abrogate LMP1-mediated inhibition of SENP2 activity. K447 is adjacent to the catalytic triad and a SENP2/SUMO interaction cluster^34^. It is also in the proximity of 19 additional lysine residues found within the C-terminal domain of SENP2. Therefore, it is possible that LMP1-induced sumoylation of SENP2 K447 regulates other C-terminal post-translational modifications of SENP2 in order to modulate SENP2 activity.

We wanted to clarify how LMP1 mediated these effects on SENP2, so due to the similarities between sumoylation and ubiquitination^1,2,51^, we switched our focus to investigate the ubiquitination of SENP2. Ubiquitinated SENP2 was detected in latently infected B-cells, but additional studies revealed that expression of LMP1 actually decreased the ubiquitination of SENP2. Treatment of cells with a known inhibitor of ubiquitination, PYR-41^52^, resulted in changes in SENP2 activity and localization that mimic those detected when LMP1 was expressed. These findings suggest that SENP2 is normally ubiquitinated and LMP1 inhibits this ubiquitination, regulating the biology of SENP2.

Of the ubiquitin-activating enzymes, ubiquitin-activating enzyme 1 (UBA1) is thought to be responsible for most of the ubiquitination within the cell^54–56^, and PYR-41 inhibits ubiquitination involving UBA1^52^. However, no changes in SENP2 turnover were observed when comparing control-treated cells to PYR-41-treated cells. PYR-41 inhibits the degradation of p53^57^, and our results showed PYR-41 treatment corresponded with loss of p53 turnover in control- and LMP1-expressing cells, which indicates a successful inhibition of UBA1 in the experiments. UBA6 is a second identified ubiquitin-activating enzyme^55^, and the effect of PYR-41 on UBA6 is known. In addition to serving as an ubiquitin-activating enzyme, UBA6 can induce a ubiquitin-like protein modification that targets a protein for degradation by the proteasome^58^. Therefore, it is possible that UBA6 is responsible for targeting SENP2 for degradation and the targeting of SENP2 by UBA6 is inhibited when LMP1 is expressed. Future directions include investigating the function of UBA6 in SENP2 biology and in LMP1-induced post-translational modifications.

The SENP2 lysine residue(s) normally post-translationally modified by ubiquitin, or non-SUMO ubiquitin-like proteins, remain unknown. SENP2 has over forty lysine residues that could be covalently modified by ubiquitin. Of these lysines, three are part of the N-terminal nuclear localization signal^34,50^. As a SUMO-protease localizing to the inner nuclear pore. SENP2 is also thought to shuttle between the nucleus and cytoplasm and aid the shuttling of select cellular proteins^50^. Once in the cytoplasm, SENP2 can be degraded by the proteasome^50^. While the half-life of SENP2 was previously determined^50^, here we confirm that SENP2 is targeted for degradation by the proteasome. LMP1 inhibits SENP2 turnover in a CTAR-dependent manner independent of SENP2 K48, which suggests that the changes in LMP1-inhibited modification of the other N-terminal SENP2 lysine residues (K28 or K60) mediated the observed changes in SENP2 biology. While identifying the amino acids that are targeted for de-ubiquitination by LMP1 expression is currently underway, the finding that these LMP1-induced changes in SENP2 levels and function happen in a sumoylation-independent manner highlights the need for additional insight into how LMP1 affects SENP2.

In summary, these data identify a new mechanism by which LMP1 modulates cellular sumoylation processes during latent EBV infections. Our previous work documented that LMP1 induces *sumo* levels, increasing the intracellular SUMO pools^40^, and hijacks Ubc9, increasing the sumoylation of cellular proteins^15^. All the while, LMP1 induces the sumoylation of SENP2 (modified at K48 and K447) and decreases the ubiquitination of SENP2, which inhibits SENP2 activity and alters SENP2 localization. The end result of targeting three distinct steps of the sumoylation process, the end result is the disruption of normal homeostasis, and the balance of protein sumoylation and de-sumoylation is tipped towards the accumulation of SUMO-modified proteins, which is detected in latently infected B-cells and in EBV-associated lymphomas^27,40^. These findings help further elucidate how LMP1 targets multiple steps of the sumoylation processes, which will influence the maintenance of viral latency, innate immune responses, and the oncogenic potential of LMP1^15,27,32^. Because the dysregulation of cellular sumoylation processes are a feature of a variety of diseases^7–15^, including EBV-associated lymphomas, understanding how LMP1 targets the different steps of the process may help identify potential new therapeutic targets to treat LMP1-expressing EBV-associated lymphomas.

## Methods and Materials

### Cells

Human embryonic kidney (HEK) 293 cells, EBV-transformed lymphoblastoid cell lines (LCLs)^27,40^, BL41 EBV negative cells^42–44^, BL41 EBV WT cells^42–44^, BL41 EBV mutant cells (P3HR1^42–44^), and Raji cells, were cultured as previously described^15,27,32,40^.

### Plasmids

LMP1 expression constructs were gifts from Nancy Raab-Traub and Wolfgang Hammerschmidt^59,60^. GFP-SENP2 and myc-SUMO-1 were obtained from Addgene.

### LMP1 knockdown

Using previously described siRNA constructs and methods, Raji cells were transiently transfected to knockdown LMP1, with a two-base mutation siRNA used as a control^61^.

### Immunoprecipitation (denaturing)

As previously described^27,32^, denaturing immunoprecipitations were performed on lysates from transfected HEK 293 cells. Immunoprecipitations were performed in the presence of 1X EDTA-free complete protease inhibitor (Santa Cruz Biotechnology) and 20 μM NEM.

### Immunoprecipitation (native)

Native immunoprecipitations were performed as previously described^27,32^.

### Western blot analysis

Immunoblotting was performed as previously described^15,27,32,40^, and bands were visualized with chemiluminescence treatment (Advansta) using the ChemiDoc Touch Imaging System (Bio-Rad).

### Protein turnover

Protein turnover and proteasome-mediated degradation was analyzed as previously described^32^. In time-course studies, cells were treated with the vehicle control (DMSO; Sigma) or cyclohexamide (Sigma; 75 μg/ml) for 4–16 hours prior to the harvesting of whole cell lysates. Densitometric analysis was performed on repeat analyses using Image J software.

### Cell compartments

Cytoplasmic, membrane, and nuclear extracts were isolated using the Qproteome cell compartment kit (Qiagen) as previously described^32^.

### Immunofluorescence microscopy

On glass coverslips, HEK 293T cells were grown and transfected. Cells were fixed, permeabilized, and stained with anti-FLAG-specific antibodies and the appropriate secondary antibodies as previously described^32,40^. FLAG-LMP1, GFP-SENP2, and DAPI expression were examined at 20X magnification using the EVOS FLoid Cell Imaging Station (Thermo Scientific). Z-stacked images were acquired at 60X magnification, with 2X zoom, using the Nikon A1 laser confocal microscope.

### Mutagenesis

Using wild-type GFP-SENP2 and site-directed mutagenesis (Stratagene) GFP-SENP2 K48R, K447R, and K48R/K447R were constructed using methods previously described^32^, with an annealing temperature of 55 °C. Resulting plasmids were sequenced to verify the mutations.

### Antibodies

FLAG-specific (M2) and actin-specific antibodies were purchased from Sigma. LMP1-specific antibodies (CS 1–4), SUMO1-specific (D-11), myc-specific (9E10), Histone H1-specific (AE-4), and GAPDH-specific (FL-335) antibodies were purchased from Santa Cruz.

### Sumo-AMC assay

Transfected HEK 293 cells were harvested, pelleted, and resuspended in 1X PBS. Four freeze-thaw cycles were administered and cell solutions were aspirated with a 22 ½ gauge needle and syringe to shear DNA. SUMO-AMC activity was performed by modifying an ubiquitin-AMC assay^62^. Fluorescence intensity was recorded using a Tecan plate reader (excitation - 345 nm, emissions - 445 nm). When applicable, fluorescence intensity was normalized to SENP2 expression levels. Samples and experiments were run in triplicate.

Similar assays were performed using equal numbers of paired BL41 cell lines and Raji cells transfected with siRNA specific for LMP1 or a control siRNA (see above).

### Sumo-Chain assay

Transfected HEK 293 cells were collected and were divided into two. One half was used for the initial time point. The second half was incubated with 2 μM preformed chains of SUMO-2 or SUMO-3 (BostonBiochem) and de-sumoylation was allowed to proceed for 30 min 37 °C. All samples were denatured in 6X SDS loading buffer and boiled for 15 minutes. Cleavage of the poly-SUMO chains was assessed by Western blot analysis.

### Nuclear envelope extraction

Nuclear envelopes were isolated from transfected cells using the Minute^(TM)^ Nuclear Envelope Protein Extraction Kit (Invent Biotechnologies Inc.) and following the manufacturer’s protocol, with the exceptions that 20 mM NEM and protease inhibitors were added to Buffers A and B prior to use.

### Statistical analysis

Unpaired, two-tailed, Student’s T-test were used for all statistical analyses. Data are shown as the means ± the standard deviation. All samples and independent experiments performed in triplicate. P-values < 0.05 were considered to be statistically significant.

## Supplementary information

Original Blots
