# Supplementary material for: The Epstein-Barr Virus Oncoprotein, LMP1, Regulates the Function of SENP2, a SUMO-protease
Source: Sci Rep. 2019 Jul 2;9:9523. doi: 10.1038/s41598-019-45825-5 (PMC6606635; doi:10.1038/s41598-019-45825-5)

The Epstein-Barr Virus Oncoprotein, LMP1, Regulates the Function of SENP2, a SUMO-protease

Thomas L. Selby, Natalie Biel, Matthew Varn, Sheetal Patel,
Akash Patel, Leslie Hilding, Ashley Ray, Tabithia Ross,
Wyatt T. Cramblet, C. Randall Moss, Angela J. Lowrey, and
Gretchen L. Bentz

Unedited blots for Figure 1: images cropped to dotted-line boxes.

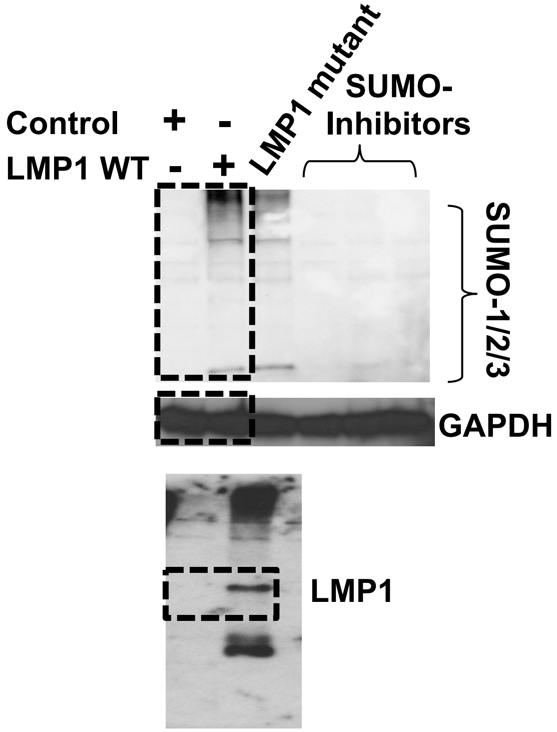

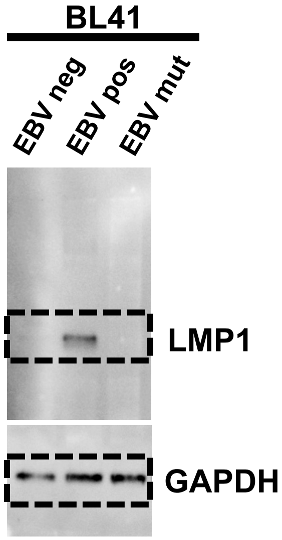

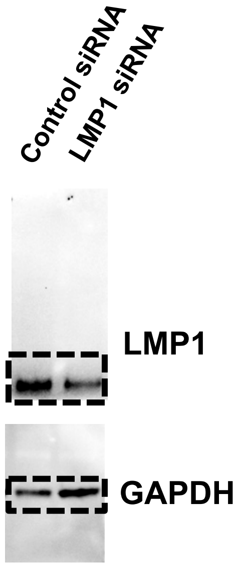

Unedited blots for Figure 2a-d: images cropped to dotted-line boxes.

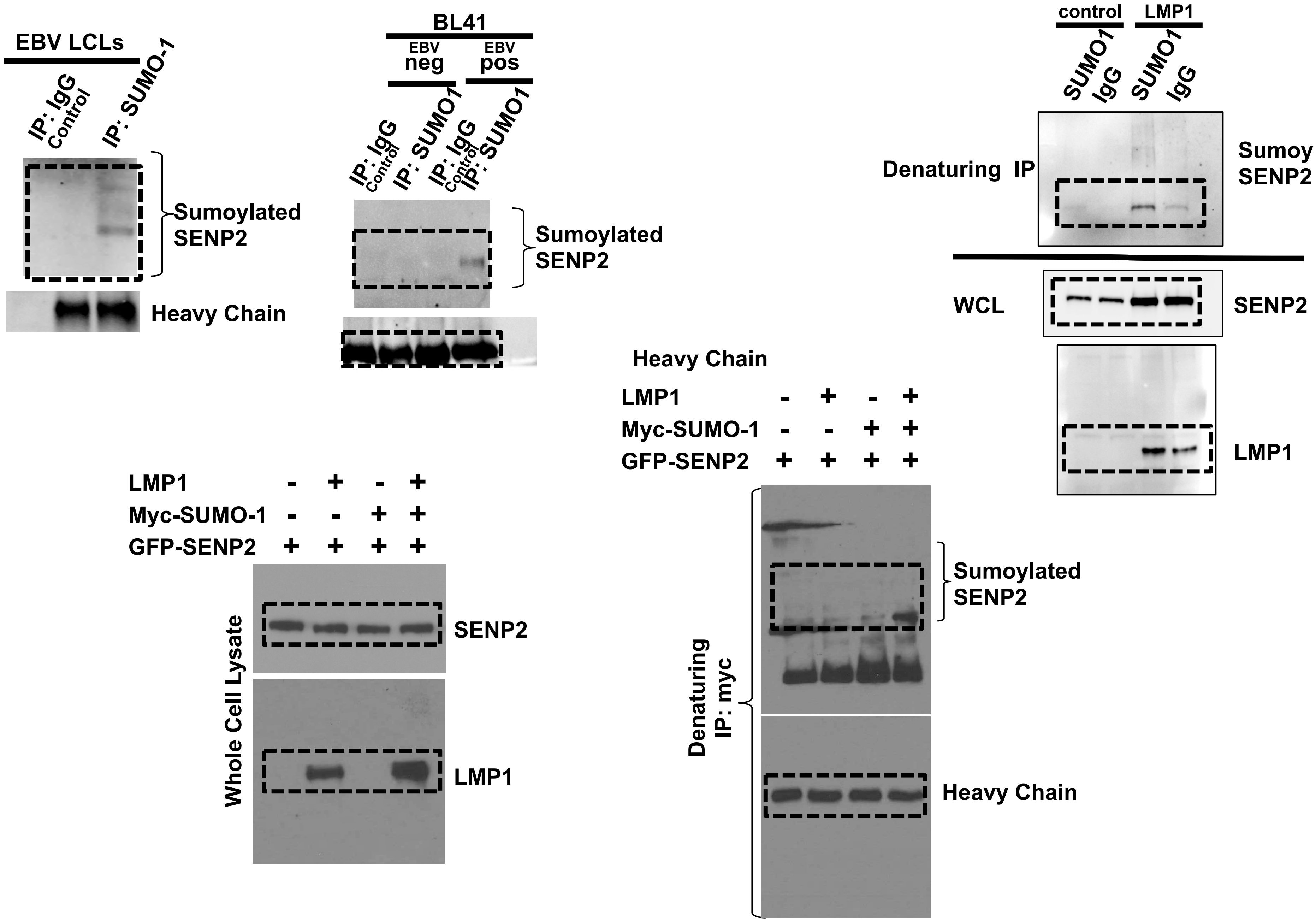

Unedited blots for Figure 3: images cropped to dotted-line boxes.

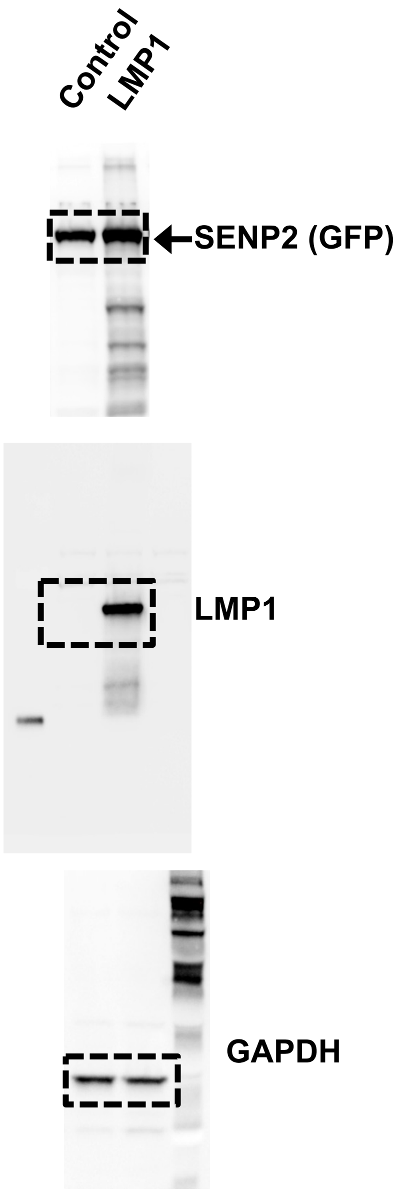

Unedited slot-blots for Figure 4a-b: images cropped to dotted-line boxes..

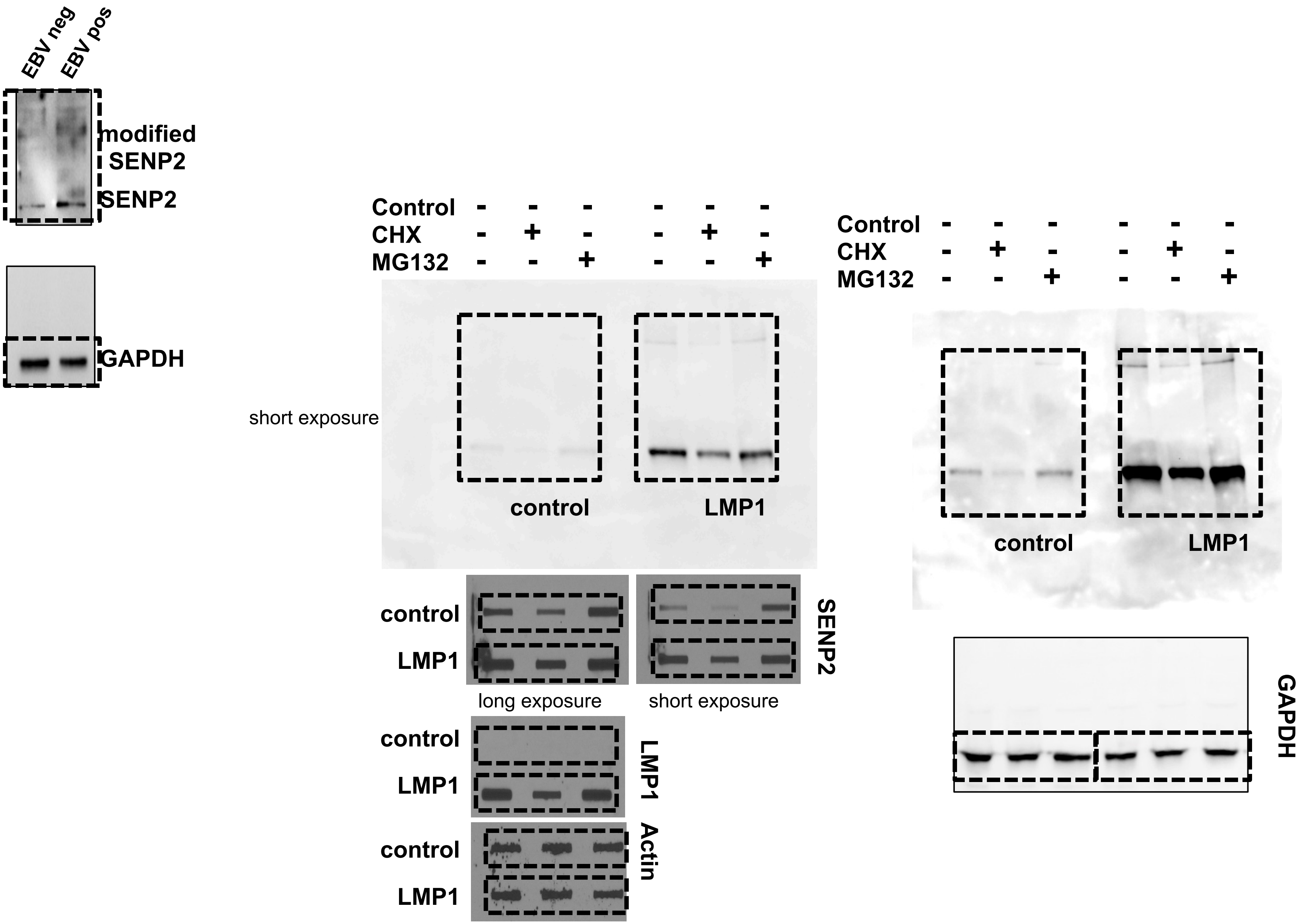

Unedited slot-blots for Figure 4d: images cropped to dotted-line boxes..

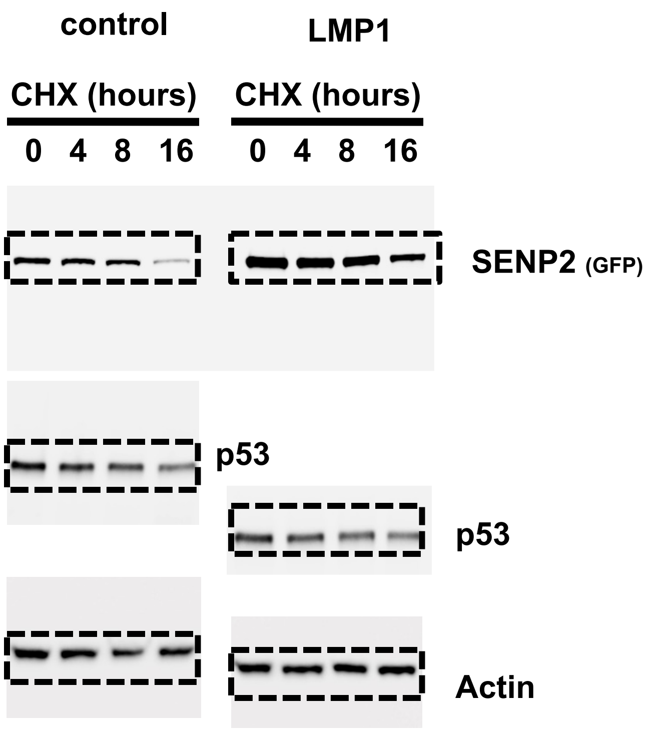

Unedited blots Figure 5: images cropped to dotted-line boxes.

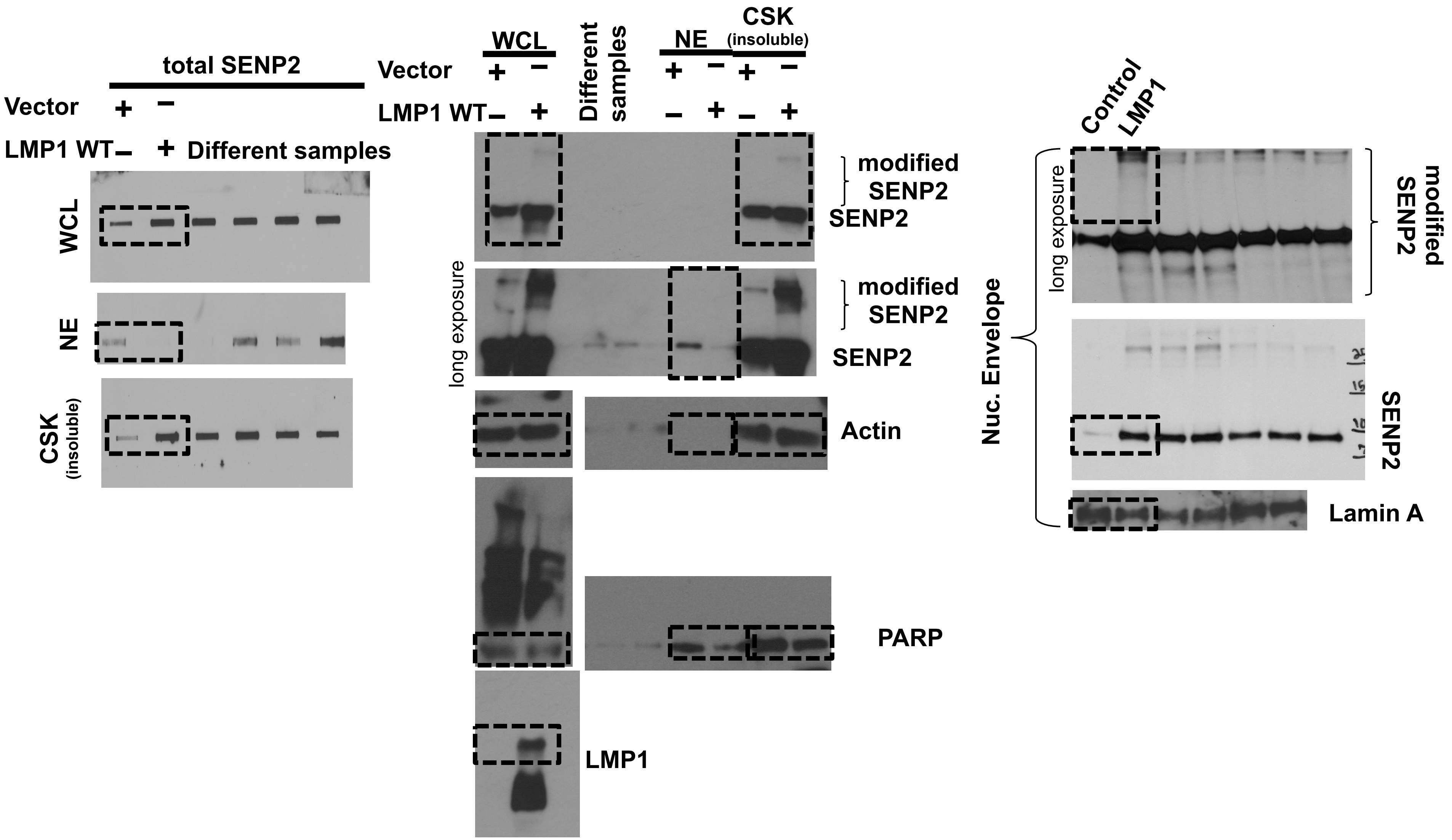

Unedited blots Figure 6: images cropped to dotted-line boxes.

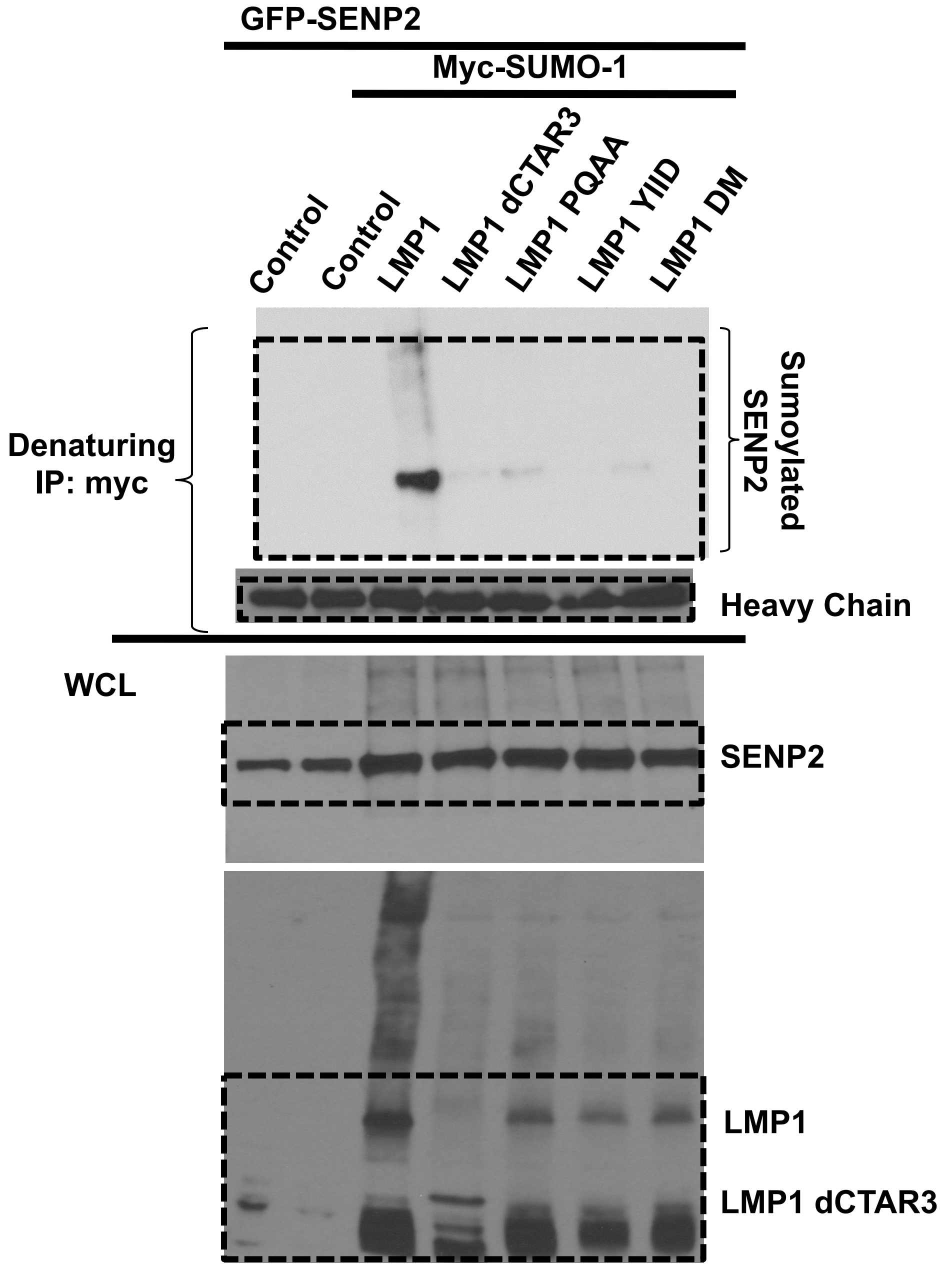

Unedited blots Figure 7: images cropped to dotted-line boxes.

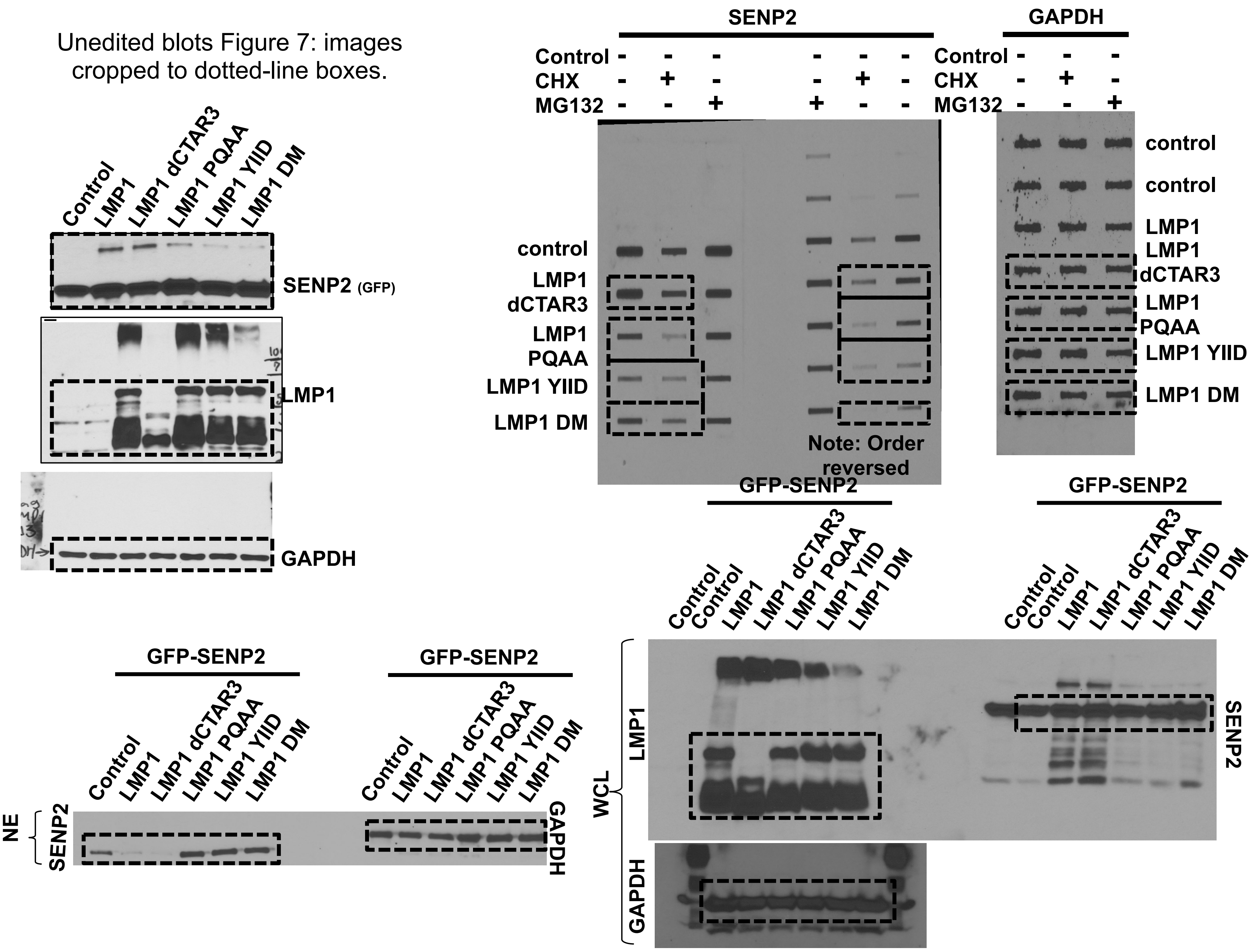

Unedited blots Figure 8: images cropped to dotted-line boxes.

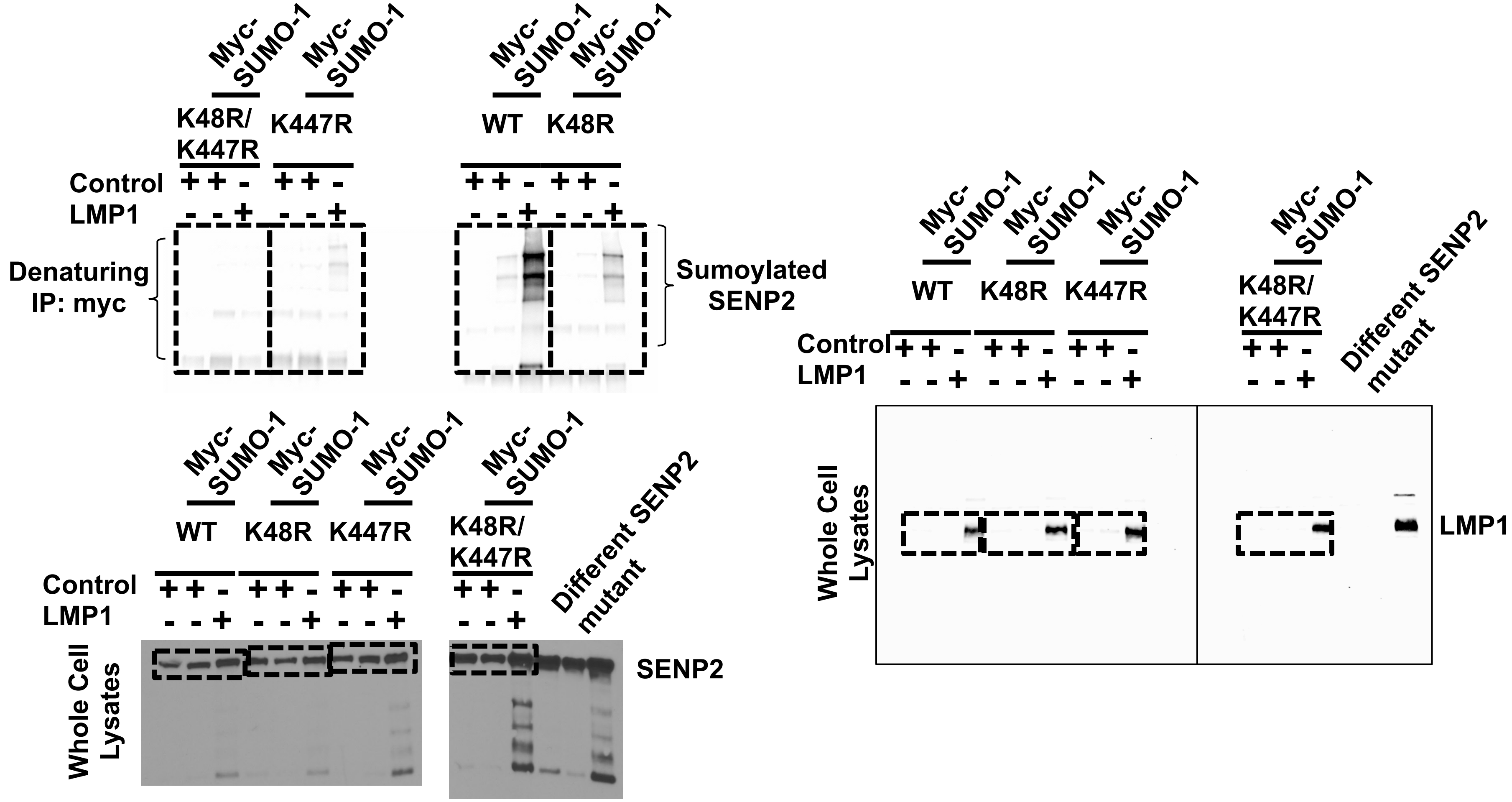

Unedited blots Figure 9a and 9c: images cropped to dotted-line boxes.

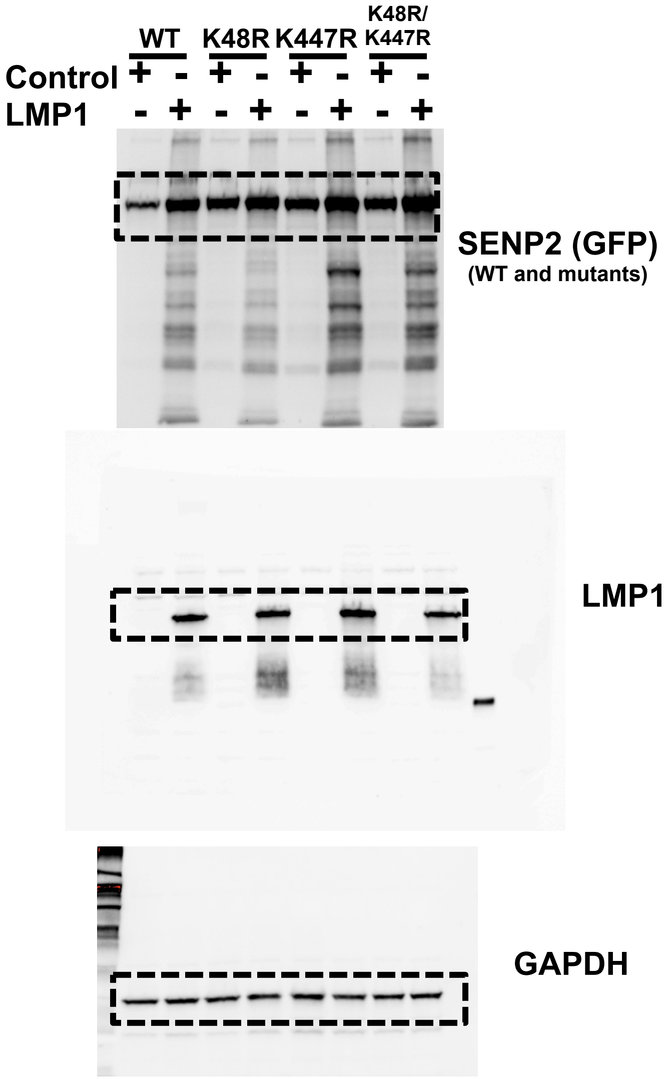

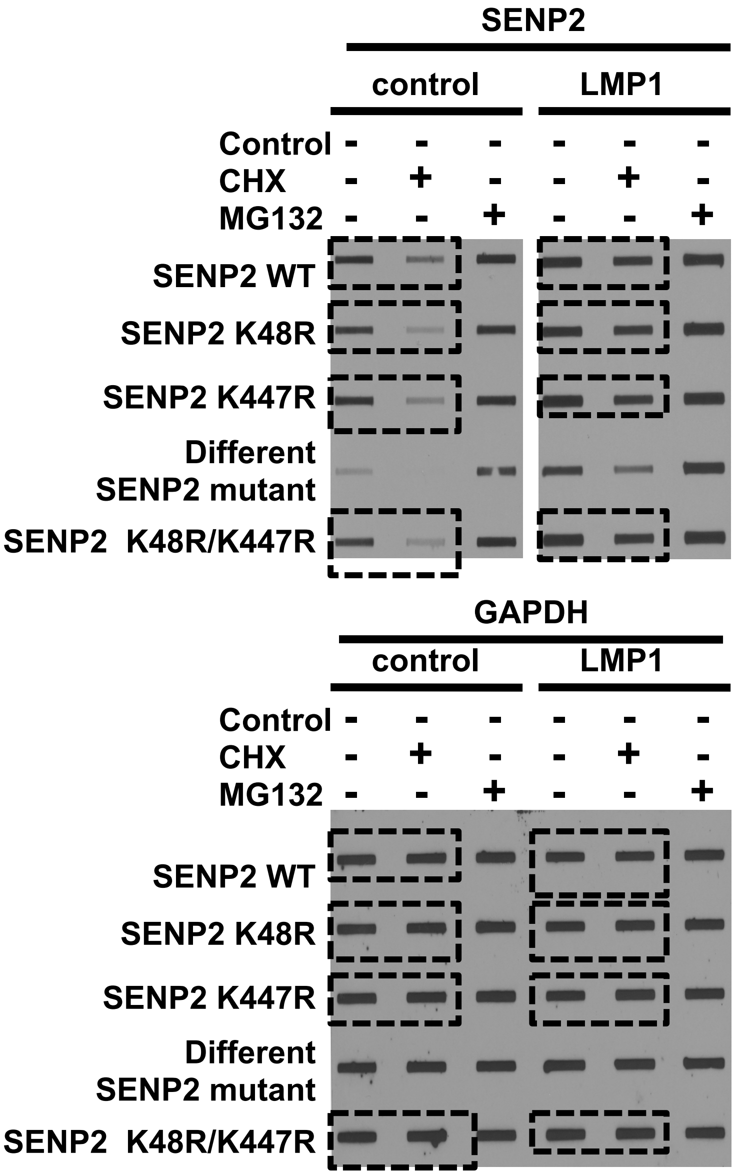

Unedited blots for Figure 9d: images cropped to dotted-line boxes.

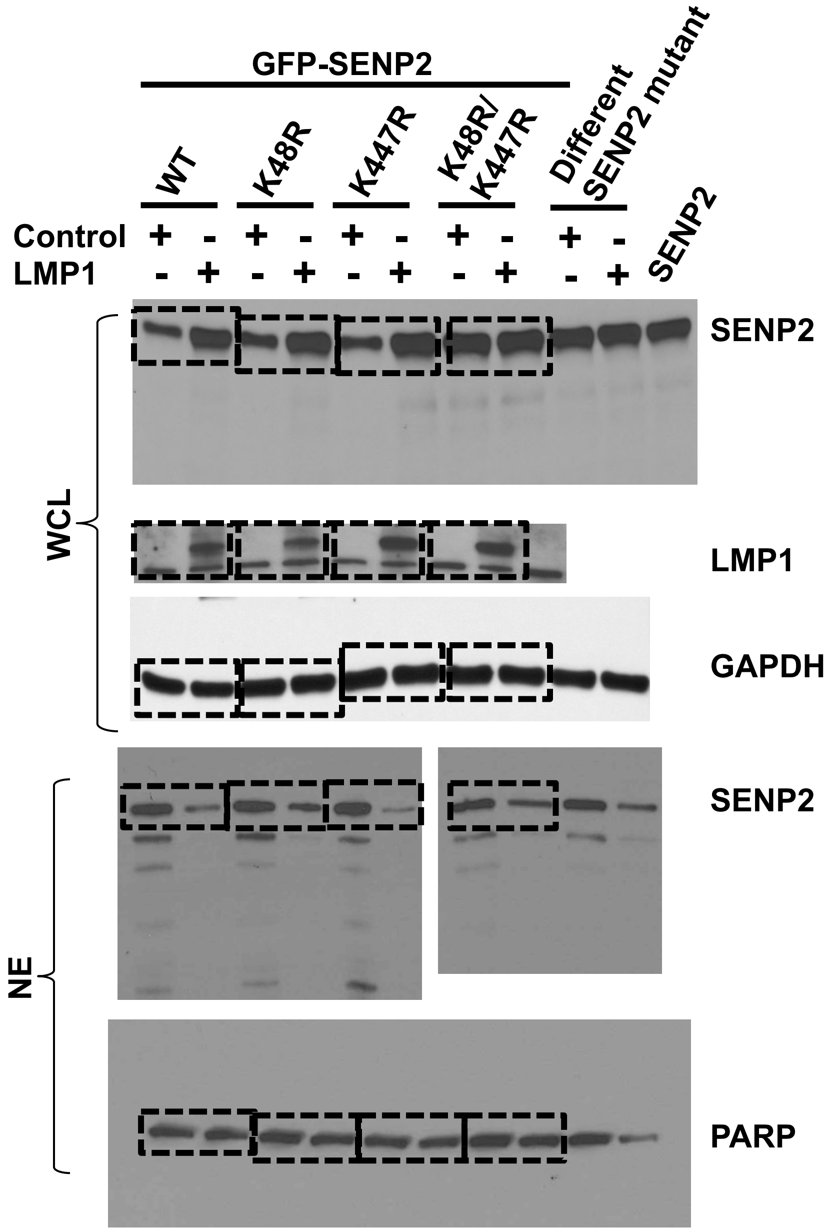

Unedited slot-blots for Figure 10: images cropped to dotted-line boxes.

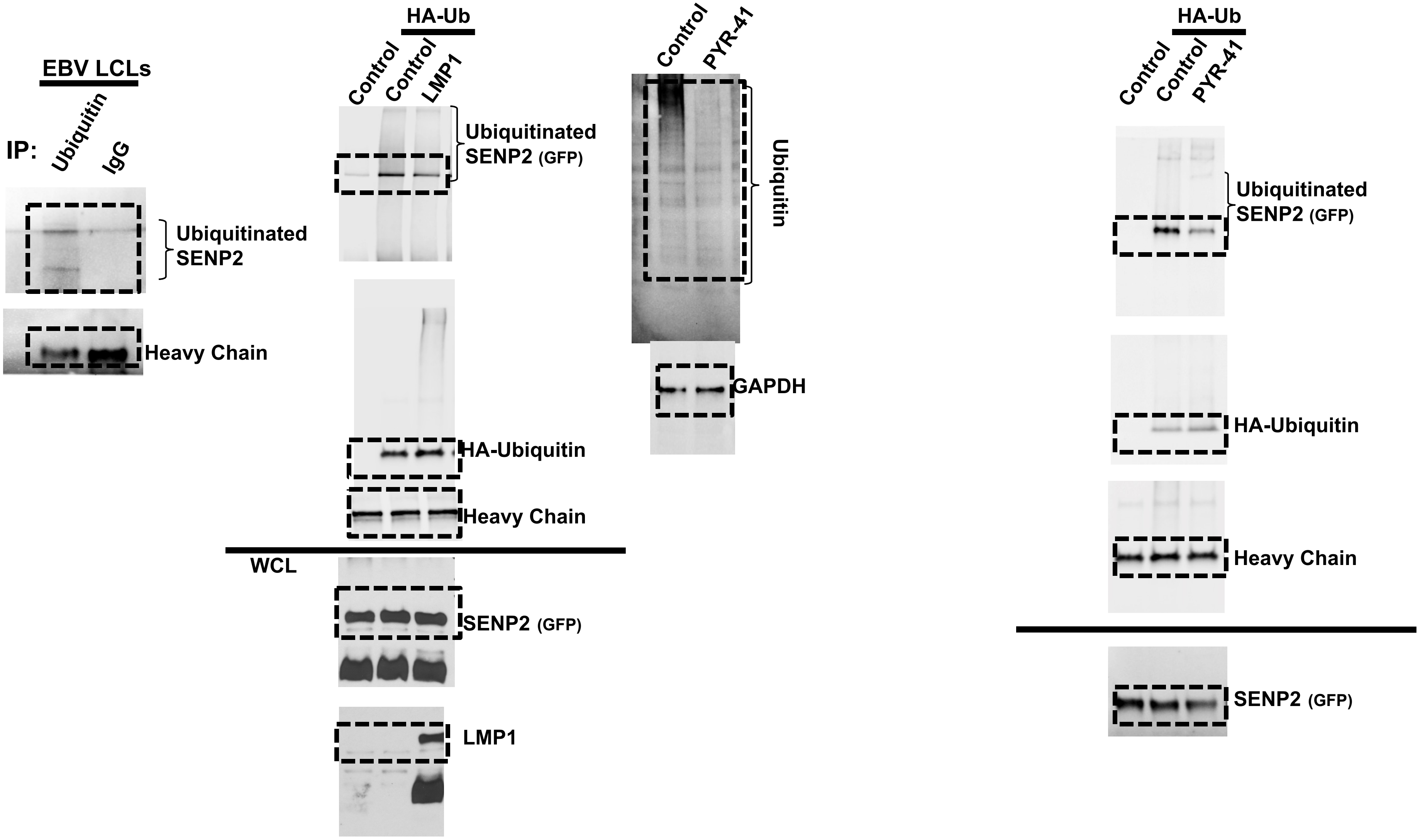

Unedited blots for Figure 11: images cropped to dotted-line boxes.

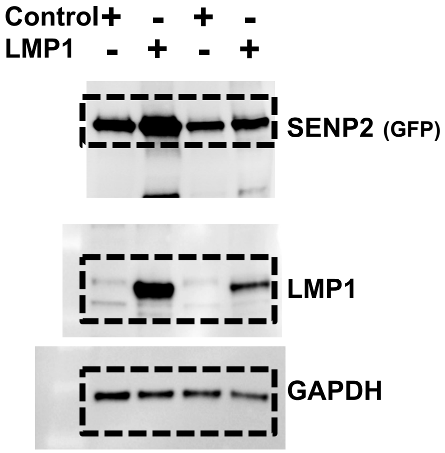

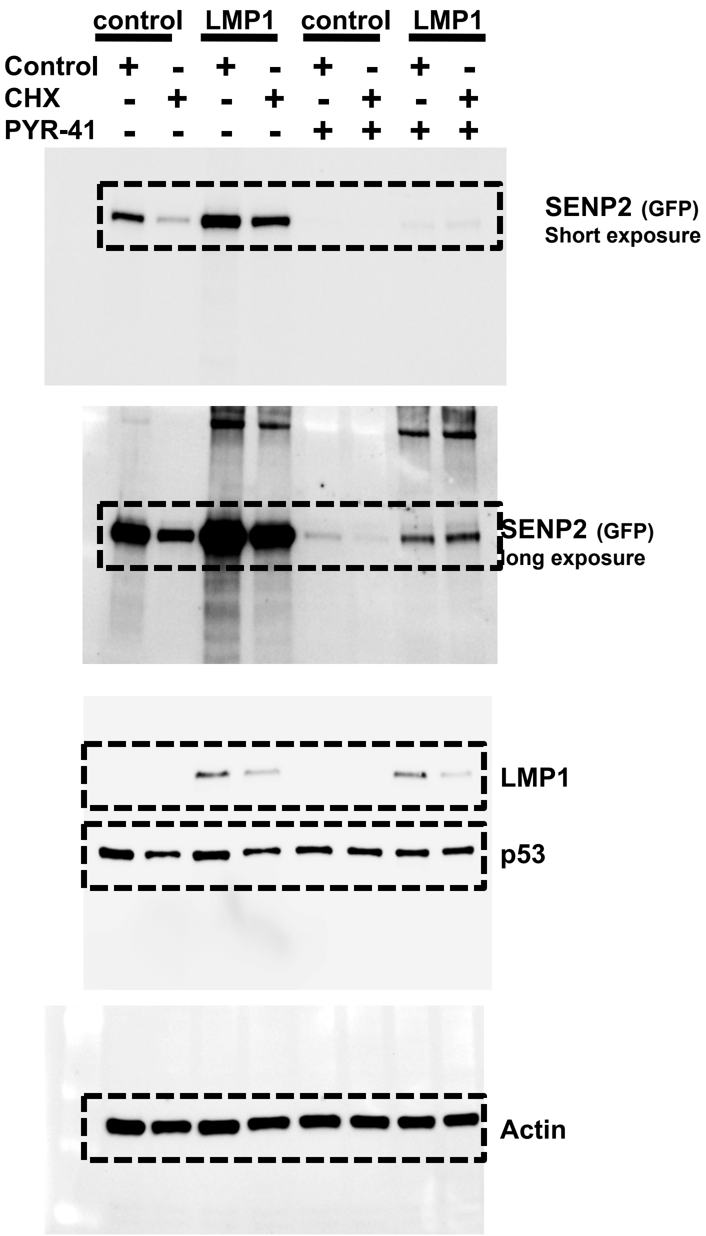

Supplement: Supplementary file 1 — Original Blots [file 41598_2019_45825_MOESM1_ESM.pdf]
